# Endophytic Fungi: An Effective Alternative Source of Plant-Derived Bioactive Compounds for Pharmacological Studies

**DOI:** 10.3390/jof8020205

**Published:** 2022-02-20

**Authors:** Juan Wen, Samuel Kumi Okyere, Shu Wang, Jianchen Wang, Lei Xie, Yinan Ran, Yanchun Hu

**Affiliations:** 1 Key Laboratory of Animal Diseases and Environmental Hazards of Sichuan Province, College of Veterinary Medicine, Sichuan Agricultural University, Chengdu 611130, China; juanwen881010@163.com (J.W.); samuel20okyere@gmail.com (S.K.O.); shuw0326@163.com (S.W.); wangjianchen01@163.com (J.W.); wsxielei@gmail.com (L.X.); ranyinan17@163.com (Y.R.); 2 Key Laboratory of Animal Disease and Human Health of Sichuan Province, Sichuan Agricultural University, Chengdu 611130, China; 3 New Ruipeng Pet Healthcare Group Co., Ltd., Shenzhen 518000, China

**Keywords:** endophytic fungi, secondary metabolites, structural feature, biological activities, drug discovery

## Abstract

Plant-associated fungi (endophytic fungi) are a biodiversity-rich group of microorganisms that are normally found asymptomatically within plant tissues or in the intercellular spaces. Endophytic fungi promote the growth of host plants by directly producing secondary metabolites, which enhances the plant’s resistance to biotic and abiotic stresses. Additionally, they are capable of biosynthesizing medically important “phytochemicals” that were initially thought to be produced only by the host plant. In this review, we summarized some compounds from endophyte fungi with novel structures and diverse biological activities published between 2011 and 2021, with a focus on the origin of endophytic fungi, the structural and biological activity of the compounds they produce, and special attention paid to the exploration of pharmacological activities and mechanisms of action of certain compounds. This review revealed that endophytic fungi had high potential to be harnessed as an alternative source of secondary metabolites for pharmacological studies.

## 1. Introduction

The term “endophytic fungi” refers to fungi that live in plant tissues throughout the entire or partial life cycle by establishing a mutually beneficial symbiotic relationship with its host plant without causing any adverse effect or disease [1,2]. They are natural components of the plant micro-ecosystem that positively affect the physiological activities of the host plant in several ways, including producing hormones such as indoleacetic acid, biosynthesizing and acquiring nutrients for plant growth and development, secreting stress-adaptor metabolites to protect the host plant from the invasion of herbivores, pathogens, and improving the host’s adaptability to abiotic stressors. In return, plants provide habitats and nutrients for endophytic fungi [3,4]. Endophytic fungi are capable of producing a rich variety of bioactive substances and can produce compounds that are identical or similar to pharmacological activities identified from plants [5]. They produce a range of metabolites of different chemical classes, including alkaloids, flavonoids, steroids, terpenoids, and phenolic compounds. Some compounds show pleiotropic and interesting pharmacological activities, including antimicrobial, antioxidant, anti-diabetic, anti-malarial, and antitumor properties. The discovery of these structurally novel and diverse active compounds provides a valuable resource for studying natural medical products from the microbiome [6,7,8]. In the search for bioactive molecules as pro-drug compounds or in the development of medicines, endophytic fungi can serve as an alternative source for valuable active plant compounds. Endophytic fungi can be harnessed to produce bioactive compounds for human pharmaceutical use when the bioactive secondary metabolites are not commercially available, derived from slow-growing or rare and endangered plants, and difficult to synthesize due to heavy molecular weight or structural complexity. Endophytic fungal secondary metabolites have drawn extensive attention among medicinal plants, mangroves, and marine microorganisms [9,10].

Endophytic fungi are a highly biodiverse and versatile microbial community that seems to be ubiquitous in nature. Studies have shown that almost all plants contain endophytic fungi, including colonized plants in the Arctic and Antarctic regions, deserts, oceans, and tropical rainforests [11,12]. They have been isolated and cultured from the roots and above-ground parts of various plants, including algae, mosses, ferns, gymnosperms, and angiosperms. Evidence from microorganism’s records in the fossil plant tissue indicated that the plant-endophytic fungal interactions have existed for approximately 400 million years, and during this time, endophytic fungi co-evolved unique biosynthetic pathways and metabolic mechanisms to synthesize complex secondary metabolites [13]. To date, only 5% of 1.5 million fungal species on Earth have been described in detail, and out of this percentage (69,000 fungal species), only 16% (11,500 species) have been cultured and studied. About 0.035–5.1 million fungal species have been found on Earth according to results from next-generation sequencing technologies [14]. Approximately 300,000 known species of higher plants exist on Earth, and each of which is a host for one or more obligate endophytic fungi. The high number of bioactive secondary metabolites found in endophytic fungi is due to their rich species diversity [15,16]. Endophytic fungi have been studied for more than 100 years, with the first endophytic strain isolated from the seeds of ryegrass (*Lolium temulentum* L.) by Vogl et al. in 1898 [17]. Stierle et al. [18] discovered the paclitaxel-producing endophytic fungus (*Taxomyces andreanae*) from the Pacific yew and then from other plant species successively. This discovery aroused the attention of mycologists and pharmaceutical chemists on endophytic fungi as a new source of bioactive substances and stimulated the interest in endophytic fungi as a sustainable source of plant metabolites. As shown in Table 1, many compounds that were isolated from endophytic fungi were also identified in some plant species as well as exhibited similar biological activity even though there were isolated from different sources, confirming endophytic fungi as an alternative source of bioactive compounds [19,20,21,22,23,24,25,26,27,28,29,30,31,32]. An overview of the recent literature surveys revealed that 51% of the bioactive substances isolated from endophytic fungi were previously unknown, with about 38% being isolated from soil microbiota [19]. Over the past decade, there has been a surge in the number of patents for endophytic fungi with new molecular secondary metabolites, which play a key role in the pharmaceutical industry, phytoremediation, and biomedicine [20,21]. Researchers are now searching for an economical, environmentally safe, and sustainable way to obtain new bioactive secondary metabolites from endophytic fungi. 

**Figure 1:**
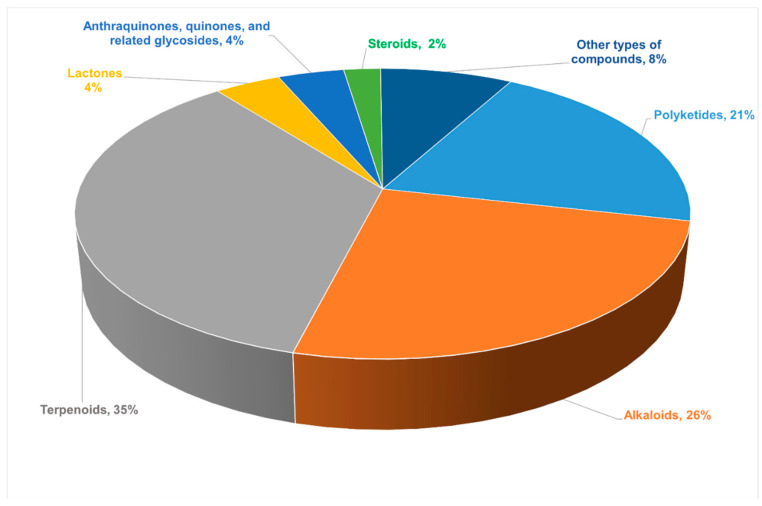
The proportion of different types of compounds among all new compounds.

This article reports 220 new compounds with rare or novel structures or skeleton structures from endophytic fungi from 82 journal articles between 2011 and 2021 and briefly describes the sources of endophytic fungi, chemical structures, and biological activities of these compounds. Among all the new compounds reported in this review, terpenoids (35%) were largest in proportion, followed by alkaloids (26%). The proportion of different types of compounds among all the new compounds are presented in Figure 1. These new compounds were obtained from different species of endophytic fungi, which had diverse chemical skeletons and exhibited diverse and interesting biological activities. Additionally, the most common pharmacological activities these compounds showed were antimicrobial and antitumor activities. However, some of the compounds showed anti-angiogenic, anti-phytotoxic, and α-glucosidase inhibitory effects. Therefore, this review summarized different insights into the prospects and challenges of endophytic fungi as an alternative source of plant-derived bioactive compounds for drug development. In addition, this review will affirm that endophytic fungi produce similar bioactive compounds just as their host plants to give knowledge for the development of drug candidates from endophytic fungi using different strategies, thus making Endophytic fungi a treasure trove of new secondary metabolites.

## 2. Bioactive New Metabolites Isolated from Endophytic Fungi and Their Biological Activities

### 2.1. Polyketides

#### 2.1.1. Chromones

**Figure 2:**
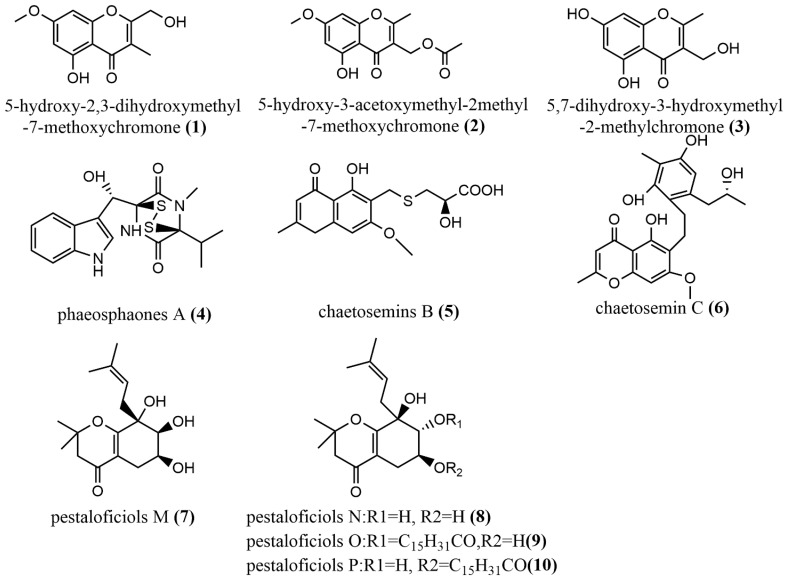
Chemical structures of chromones.

The induction of endophyte metabolism by adding Host components was used to add the same phytocomponents (2R, 3R)-3, 5, 7- trihydroxyflavanone 3-acetate in Botryosphaeria ramosa L29 potato dextrose broth culture to induce the production of 5-hydroxy2,3-dihydroxymethyl-7-methoxychromone **1** (Figure 2), 5-hydroxy-3-acetoxymethyl-2-methyl-7- methoxychromone **2** (Figure 2) and 5,7-dihydroxy-3-hydroxymethyl-2-methylchromone **3** (Figure 2), where Compounds **1**–**3** displayed acceptable antimicrobial activities against *Fusarium oxysporum* with MIC values of 50 μg/mL, 50 μg/mL, and 6.25 μg/mL, respectively. These values were superior compared to those of the positive drug—triadimefon—for the antimicrobial test (with an MIC value of 100 μg/mL) [36]. This indicated that the induction of endophytes metabolism to produce bioactive components of interest might be an ideal strategy for easy identification of drug candidates from these microbes; however, there is the need for long-term studies on how specific components influence endophytes metabolism and the bioactive compounds there are linked with. Phaeosphaonesa A **4** (Figure 2), isolated from *Phaeosphaeria fuckelii*, contains a β-(oxy)thiotryptophan motif structure that is rare in nature. Compound **4** showed stronger inhibition activity of mushroom tyrosinase than the positive control kojic acid (IC_50_ value of 40.4 μM) at 100 μM concentration, with an IC_50_ value of 33.2 μM [37]. Two aromatic chromones, Chaetosemins B–C **5**–**6** (Figure 2), were isolated from *Chaetomium seminudum* brown rice cultures, and compounds **5**–**6** contained L-cysteine and D-cysteine units, respectively. Compound **5** showed antifungal activity against *Magnaporthe oryzae* and *Gibberella saubinetti*, with MIC values of 6.25 μM and 12.5 μM, respectively. Compound **6** showed significant antioxidant activity at a concentration of 50 μM with a DPPH radical scavenging rate of 50.7% [38]. Pestaloficiols M–P **7**–**10** (Figure 2), which are new isoprenylated chromone derivatives, were isolated from brown rice culture extract of the plant endophytic fungus *Pestalotiopsis fici*. The structures of these compounds were elucidated primarily by MS and NMR techniques. Compounds **7**–**8** displayed inhibitory effects on HIV-1 replication in C8166 cells, with EC_50_ values of 56.5 μM and 10.5 μM, respectively (the EC _50_ value of the positive control Indinavir Sulfate was 8.2 μM), whereas compounds **9**–**10** showed cytotoxic activity against the human tumor cell line HeLa, with IC_50_ values of 56.2 μM and 74.9 μM, respectively (the positive control 5-fluorouracil has an IC_50_ of 10.0 μM). Compound **10** exhibited a potent antifungal activity against *Aspergillus fumigatus* at IC_50_ = 7.35 μM) [39]. 

#### 2.1.2. α-Pyrones

**Figure 3:**
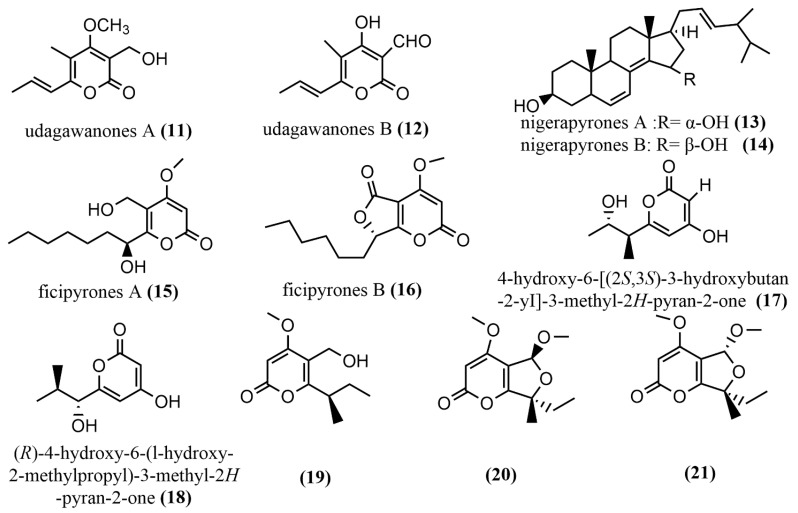
Chemical structures of α-pyrone compounds.

Two tetrasubstituted α-pyrone derivatives—*Neurospora udagawae* udagawanones A-B **11**–**12** (Figure 3)—were isolated from oak endophytic fungi, with both containing unique oxidation functional groups at the C-2 position. Compound **11** exhibited potent antifungal activity against *Rhodoturula glutinis* with MIC = 66 μg/mL*)*. Additionally, compounds **11** and **13** showed moderate cytotoxic activity against KB3.1 cells with IC_50_ = 27 μg/mL [40]. The study revealed moderate activity of compounds **11** and **12** against fungi and mammalian cells, and this may be as a result of the method (serial dilution antimicrobial assay) used; therefore, it is suggested that other biological tests be employed to verify these findings. The nigerapyrones A–B **13**–**14** (Figure 3) were obtained from *Aspergillus niger* MA-132, which was isolated from the mangrove plant *Avicennia marina*. Compounds **13**–**14** both showed potent antifungal activities against two tumor cell lines (HL60 and A549), with IC_50_ values ranging from 0.3 to 5.41 μM [41]. The ficipyrones A–B **15**–**16** (Figure 3) were isolated from solid cultures of *Pestalotiopsis fici*. Compound **15** showed significant antifungal activity against *Gibberella zeae* CGMCC 3.2873, with an IC_50_ value of 15.9 μM, but had no activity against *Fusarium culmorum* CGMCC 3.4595 and *Verticillium aiboatrum* CGMCC 3.4306 [42]. The endophytic fungus *Aspergillus oryzae* was isolated from the rhizome of *Paris polyphylla* in Dali, Yunnan, China, and 4-hydroxy-6-[(2S, 3S)-3-hydroxybutan-2-yI]-3-methyl-2H-pyran-2-one **17** (Figure 3) and (R)-4-hydroxy-6-(l-hydroxy-2-methylpropyl)-3-methyl-2H-pyran-2-one **18** (Figure 3) were obtained from this fungi. However, the biological activities of these compounds were not tested in the study; hence, investigating the biological activities of these compounds is needed, as it may yield a very important source of drug activity [43].The pyran-2-one scaffold compounds **19**–**21** (Figure 3) were isolated by adding 10 mg/L DNA methyltransferase inhibitor 5-aza-2-deoxycytidine to *Penicillium herquei* liquid cultures, whereas the MTT method was used to measure the cytotoxicity of all compounds in MDA-ME-231 and MV-411 cell lines. Compounds **19**–**21** showed weak cytotoxicity only against the MV4-11 cell line with IC_50_ values of 90.09 µM, 74.16 µM, and 70.00 µM, respectively [44].

#### 2.1.3. Other Polyketides

**Figure 4:**
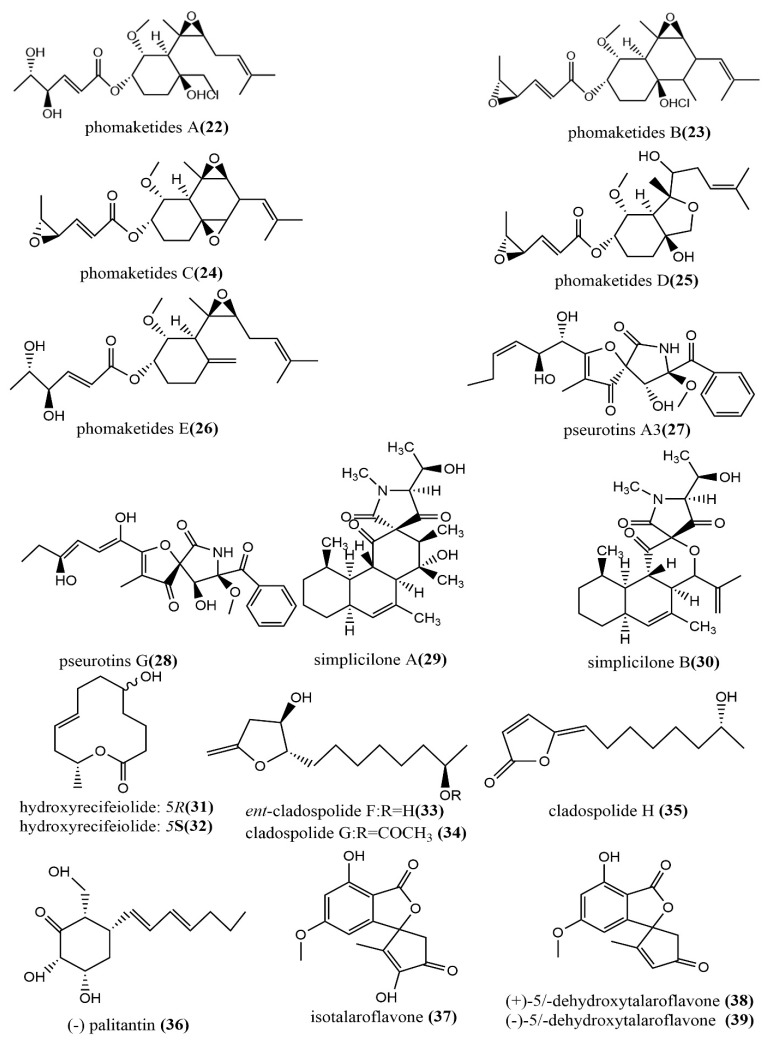

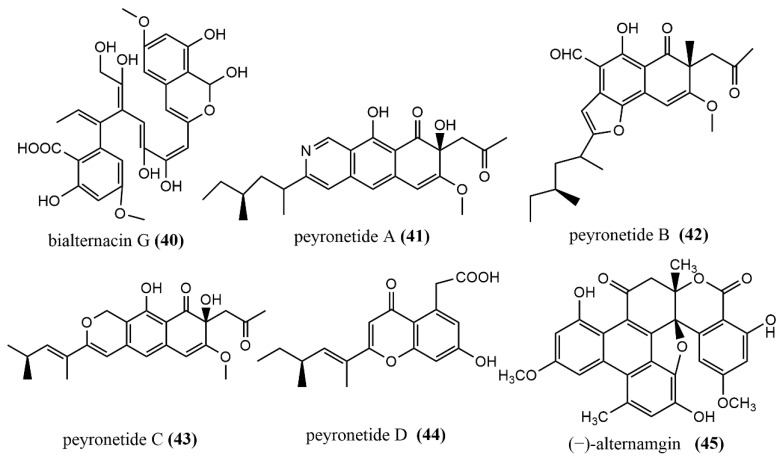
Chemical structures composition of other polyketides.

The phomaketides A–E **22**–**26** (Figure 4), pseurotins A_3_ **27** (Figure 4), and pseurotins G **28** (Figure 4) were isolated from fermentation broth and mycelial extracts of the marine red algae endophytic fungus *Phoma* sp. NTOU4195. The mouse macrophages RAW264 were induced using the endothelial progenitor cells of human umbilical cord blood, lipopolysaccharide (LPS), to assess the anti-angiogenic and anti-inflammatory activities of all compounds. Compound **22** showed potent anti-angiogenic activity by inhibiting endothelial cell proliferation, with an IC_50_ value of 8.1 μM. Compound **24** at the concentration of 20 μM induced effective nitric oxide (NO) inhibition activity against LPS-induced RAW264.7 cells, with an IC_50_ value of 8.8 μM [45]. There were two tetracyclic polyketide compounds, simplicilone A–B **29**–**30** (Figure 4), containing helical centers obtained from the broth culture of the endophytic fungus *Simplicillium* sp., which was isolated from the bark of the medicinal plant *Duguetia staudtii* (Engl. and Diels) Chatrou in the Cameroon region. Compounds **29**–**30** showed weak cytotoxic activities against the KB3.1 cell line, with IC_50_ values of 1.25 μg/mL and 2.29 μg/mL, respectively, but had no antimicrobial activity against the tested bacteria (*Staphylococcus aureus* DSM 346 and *Bacillus subtilis* DSM 10) [46]. 5R-hydroxyrecifeiolide **31** (Figure 4), 5S-hydroxyrecifeiolide **32** (Figure 4), and ent-cladospolide F–H **33**–**35** (Figure 4) were also isolated from the endophytic fungal strain *Cladosporium cladosporioides* MA-299, which was obtained from the leaves of the mangrove plant *Bruguiera gymnorrhiza* from Hainan Island, China. Compounds **31**–**35** showed potent antimicrobial activities against *Escherichia coli* and *Staphylococcus aureus*, with MIC values ranging from 1.0 to 64 μg/mL. Compound **33** showed moderate inhibition activity against acetylcholinesterase, with an IC_50_ value of 40.26 μM [47]. The antimicrobial polyketide compound, palitantin **36** (Figure 4), was obtained from *Aspergillus fumigatiaffnis* and isolated from healthy leaves of *Tribulus terrestris L.* In addition, compound **36** showed effective antimicrobial activity against the multi-drug-resistant pathogens *Enterococcus faecalis* UW 2689 and *Streptococcus pneumoniae* 25697, both with an MIC value of 64 μg/mL [48]. The four polyketide derivatives—isotalaroflavone **37** (Figure 4), (+/−)-50-dehydroxytalaroflavone **38**–**39** (Figure 4), and bialternacin G **40** (Figure 4)—were obtained from the endophytic fungus *Alternaria alternata* ZHJG5 isolated from the leaves of *Cercis chinensis*, which was collected from the Nanjing Botanical Garden, Nanjing, China. They exhibited potent antimicrobial activity against *Xanthomonas oryzae* pv. *oryzicola (Xoc)* and *Ralstonia solanacearum*, with MIC values ranging from 0.5 to 64 μg/mL. Compound **37** at the concentration of 200 μg/mL showed a significant protective effect against the bacterial blight of rice caused by *Xanthomonas oryzae* pv. *oryza*, with a protection rate of 75.1% [49]. Four polyketide derivatives containing the benzoisoquinoline-9-one moiety structure peyronetides A–D **41**–**44** (Figure 4) were isolated from the mycelial crude acetone extract of *Peyronellaea* sp. FT431. Compounds **41**–**42** showed moderate to weak cytotoxic activity against human kidney cancer cell line TK10 and human ovarian cancer cell line A2780cisR, with IC_50_ values ranging from 6.7 to 29.2 μM [50]. The aromatic polyketide compound, (−)alternamgin **45** (Figure 4), was obtained from potato dextrose broth cultures of the endophytic fungus *Alternaria* sp. MG1 isolated from *Vitis quinquangularis*. This compound was of particular interest because it had the rare dibenzopyrone functionality of 6/6/6/6/5/6/6/6 heptacyclic backbone. Compound **45** displayed a weak cytotoxic activity against cells from two tested cell lines (Hela and HepG2), both with IC_50_ values exceeding 20 μM [51].

In summary, Polyketides, such as chromones and α-pyrone, and their derivatives identified from plant sources have also been found in endophytic fungi in recent studies. Chromones and their derivatives isolated from both plant and endophytic fungi sources all showed antimicrobial properties against specific pathogens; therefore, chromones from endophytic fungus can be used in the development of antimicrobials in the place of plant chromones to reduce the depletion of plants’ resources in the ecosystem.

### 2.2. Alkaloids

#### 2.2.1. Cytochalasin

**Figure 5:**
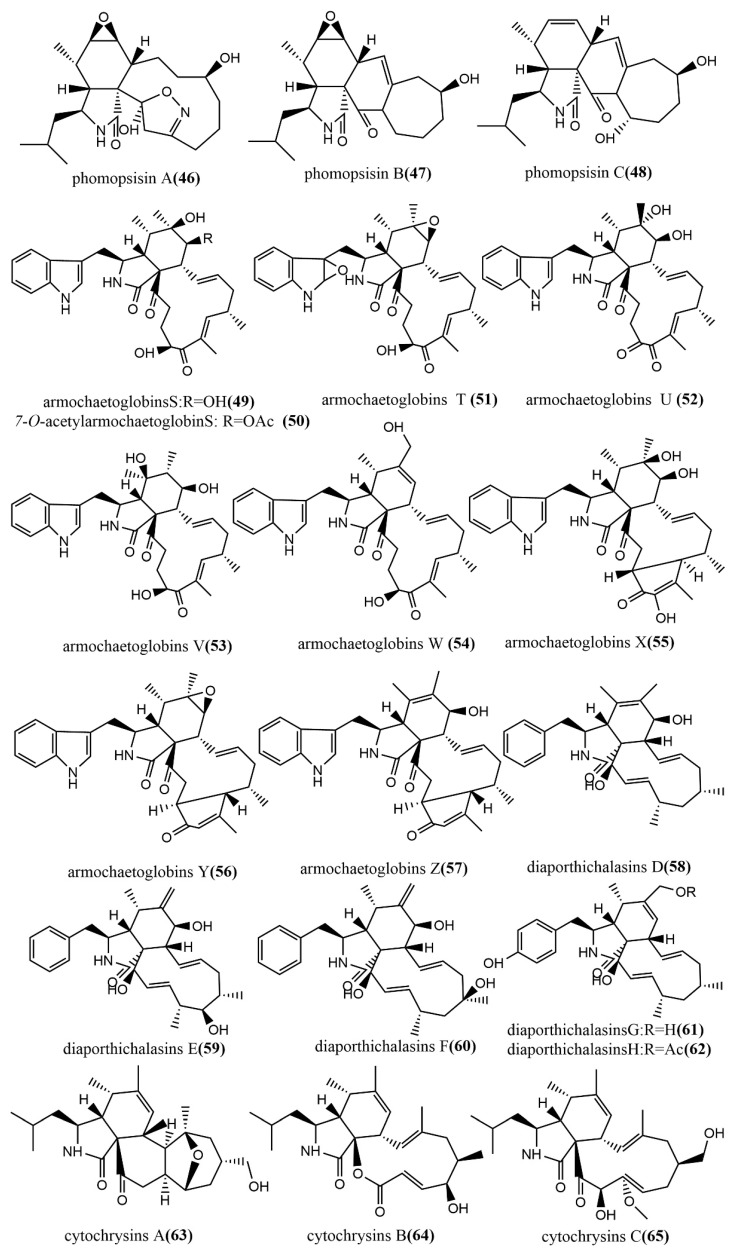
Chemical structures composition of cytochalasins.

The methylation-deficient backbone, Phomopsisin A–C **46**–**48** (Figure 5), was obtained from brown rice cultures of *Phomopsis* sp. sh917, which was isolated from *Isodon eriocalyx var. laxiflora* stems. Compound **46** contained an unusual 5/6/11/5 tetracyclic ring system 2H-isoxazole moiety and showed significant inhibition activity against LPS-induced NO production in RAW264.7 cells, with an IC_50_ value of 32.38 μM, which was more potent than the positive control L-NMMA (IC_50_ value of 42.34 μM) [52]. The highly oxidized cytochalasin alkaloids—armochaetoglobins S–Z **49**–**57** (Figure 5) and 7-O-acetylarmochaetoglobin S **50** (Figure 5)—were identified and isolated from *Chaetomium globosum* TW1-1. The effects of all compounds on five tested human cancer cell lines (HL-60, A-549, SMMC-7721, MCF-7, and SW-480) were measured using the MTT method. Compounds **56**–**57** showed potent cytotoxic activities, with IC_50_ values ranging from 10.45 to 30.42 µM [53]. Furthermore, diaporthichalasins D–H **58**–**62** (Figure 5) were obtained from solid cultures of the endophytic fungus *Diaporthe* sp. SC-J0138 isolated from the leaves of the pteridophyte *Cyclosorus parasiticus*, and the MTS method was used to evaluate the cytotoxic activities of these compounds on four human cancer cell lines (A549, HeLa, HepG2, and MCF-7). Compound **58** exhibited significant cytotoxic activity against all tested human cancer cell lines; compounds **59**–**62** exhibited selective cytotoxic activities against some cell lines [54]. Cytochrysins A–C **63**–**65** (Figure 5) were obtained from rice cultures of *Cytospora chrysosperma* HYQZ-931, an endophytic fungus isolated from the desert plant *Hippophae rhamnoides.* Compound **63** showed significant antimicrobial activity to *Enterococcus faecium*, with an MIC value of 25 μg/mL. Compound **65** showed potent antimicrobial activity to *Staphylococcus aureus,* with an MIC value of 25 μg/mL [55].

#### 2.2.2. Indole Alkaloids 

**Figure 6:**
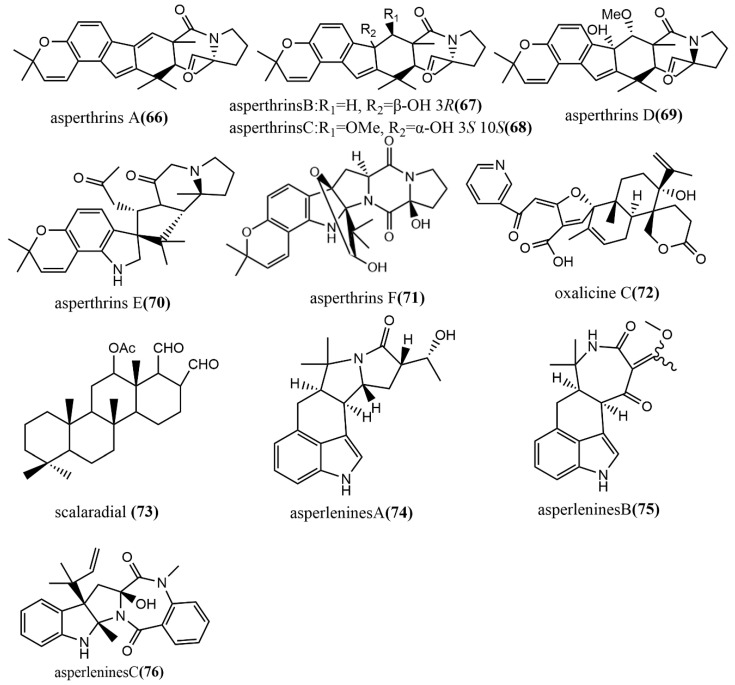
Chemical structures of indole alkaloids.

Six prenylated indole alkaloids, asperthrins A–F **66**–**71** (Figure 6), were derived from the marine endophytic fungus *Aspergillus* sp. YJ191021. Compound **66** showed moderate antimicrobial activity against *Vibrio anguillarum*, with an MIC value of 8 μg/mL. Additionally, the compounds **66** and **69** showed potent–weak anti-inflammatory activities against propionibacterium acnes-induced human mononuclear cell line (THP-1), with IC_50_ values of 1.46 μΜ and 30.5 μΜ, respectively, while compound **66** showed higher anti-inflammatory activity than the positive control Tretinoin at an IC_50_ value of 3.38 μM [56]. The α-pyrone meroterpenoid-type alkaloid, oxalicine C **72** (Figure 6), was obtained from *Penicillium chrysogenum* XNM-12, which was isolated from the marine brown algae *Leathesia nana*. Compound **72** showed potent antimicrobial activity against the phytopathogenic fungus *Ralstonia solanacearum*, with an MIC of 8 μg/mL [57]. Scalarane **73** (Figure 6) was isolated from *Hypomontagnella monticulosa* Zg15SU through the potato dextrose liquid culture. Compound **73** showed potent cytotoxic activity against cancer cell lines Panc-1, NBT-T2, and HCT116, with IC_50_ values of 0.05, 0.75, and 0.05 μg/mL, respectively [58]. Asperlenines A–C **74**–**76** (Figure 6) were isolated from *Aspergillus lentulus* DTO 327G5 cultures, and the antimicrobial activity of all compounds was evaluated using the broth-microdilution method against five tested agricultural pathogens (*Xanthomonas oryzae* pv. *Oryzae*, *Xanthomonas oryzae* pv. *Oryzicola*, *Rhizoctonia solani*, *Fusarium oxysporum*, and *Colletotrichum gloeosporioides)*. Compounds **74**–**76** showed moderate to weak antimicrobial activities against *Xanthomonas oryzae* pv. *Oryzae* and *Xanthomonas oryzae* pv*. Oryzicola*, with MIC values ranging from 25 to 100 μg/mL [59].

#### 2.2.3. Diketopiperazine Derivatives 

**Figure 7:**
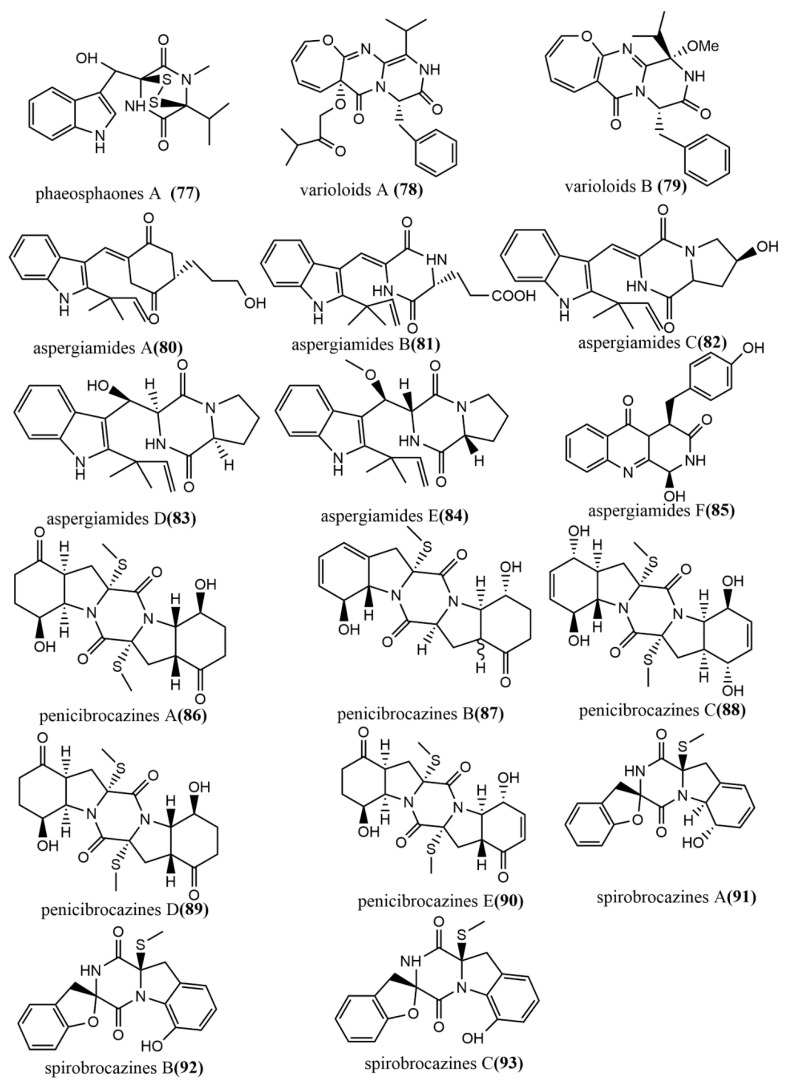
Chemical structures of diketopiperazine derivatives.

The thiodiketopiperazine alkaloid, phaeosphaones D **77** (Figure 7), featuring an unusual β-(oxy) thiotryptophan motif, was obtained from endophytic fungus *Phaeosphaeria fuckelii* isolated from the medicinal plant *Phlomis umbrosa.* Compound **77** showed stronger mushroom tyrosinase inhibition activity than the positive control kojic acid (IC_50_ value of 40.4 μM), with an IC_50_ value of 33.2 μM. [60]. The oxepine-containing diketopiperazine-type alkaloids, varioloids A-B **78**–**79** (Figure 7), were obtained from *Paecilomyces variotii* EN-291, which was isolated from the marine red alga *Grateloupia turuturu*. Compounds **78**–**79** showed potent antifungal effects against *Fusarium graminearum*, with MIC values of 8 μg/mL and 4 μg/mL, respectively [61]. Aspergiamides A–F **80**–**85** (Figure 7) were isolated from the endophytic fungus *Aspergillus* sp. 16-5 of mangroves, and all compounds were evaluated for their inhibition activities against protein-tyrosine phosphatase 1B (PTP1B) and α-glucosidase. Compounds **80** and **81** showed potent to moderate α-glucosidase inhibition activities, with IC_50_ values of 18.2 µM and 40.7 µM, respectively. Compounds **80**–**85** did not show significant PTP1B inhibition activities (<10% inhibition) at 100 µg/mL [62]. Five sulfide diketopiperazines derivatives, penicibrocazines A–E **86**–**90** (Figure 7), were obtained from the endophytic fungus *Penicillium brocae* MA-231 isolated from the mangrove plant *Avicennia marina*. The antimicrobial effects of all compounds were evaluated by the agar diffusion method against five tested pathogens (*Aeromonas hydrophilia*, *Escherichia coli*, *Staphylococcus aureus*, *Vibrio arveyi*, and *V. parahaemolyticus*). Compounds **86**–**90** showed potent antimicrobial activities against *S. aureus*, with MIC values ranging from 0.25 to 32 μg/mL [63]. Spirobrocazines A–C **91**–**93** (Figure 7) were isolated from the mangrove-derived *Penicillium brocae* MA-231. Compounds **91**–**93** contained a 6/5/6/5/6 cyclic system with a rare spirocyclic center at C-2. All compounds showed moderate antimicrobial activities against *S. aureus*, *Aeromonas hydrophilia*, and *Vibrio harveyi*, with MIC values ranging from 16 to 64 μg/mL [64].

#### 2.2.4. Other Types of Alkaloids

**Figure 8:**
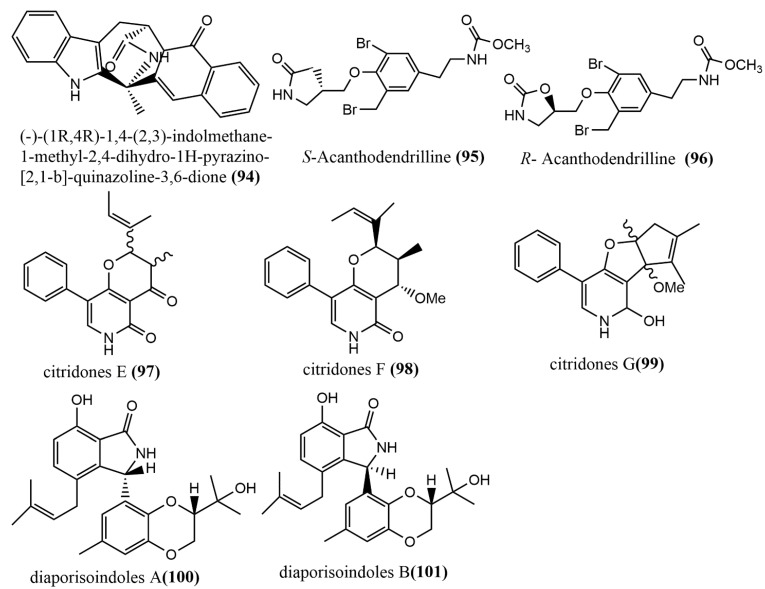
Chemical structure of other types of alkaloids.

The quinazoline alkaloid (-)-(1R,4R)-1,4-(2,3)-indolmethane-1-methyl-2,4-dihydro-1H-pyrazino-[2,1-b]-quinazoline-3,6-dione **94** (Figure 8) was obtained from the endophytic fungus *Penicillium vinaceum* X1, which was isolated from corms of *Crocus sativus* (Iridaceae). The in vitro cytotoxicity of compound **94** was evaluated against three human tumor cell lines (A549, LOVO, and MCF-7), to which compound **94** showed weak cytotoxic activities against all human tumor cell lines, with IC_50_ values of 76.83, 68.08, and 40.55 μg/mL, respectively [65]. The enantiomeric bromotyrosine alkaloids S-Acanthodendrilline **95** (Figure 8) and R-Acanthodendrilline **96** (Figure 8) were isolated from the ethyl acetate extract of the sponge endophytic fungus *Acanthodendrilla* sp. The cytotoxic activities of compounds **95**–**96** against human non-small cell lung cancer H292 and normal human immortalized fibroblast HaCaT cell lines were evaluated using the MTT method. Compound **95** (IC_50_ value of 58.5 µM) was approximately three times more potent than compound **96** (IC_50_ value of 173.5 µM) against the H292 cell line. Compounds **95**–**96** exhibited efficient and selective cytotoxic activities against H292 and HaCaT cell lines, with IC_50_ values ranging from 58.5 to 173.5 µM and >400 µM, respectively [66]. Three phenylpyridone derivatives, citridones E–G **97**–**99** (Figure 8), were obtained from the endophytic fungal strain *Penicillium sumatrense* GZWMJZ-313 9, which was isolated from the leaves of *Garcinia multiflora*. These compounds showed moderate to weak antimicrobial activities against *Staphylococcus aureus* ATCC6538, *Pseudomonas aeruginosa* ATCC10145, and *Escherichia coli* ATCC11775, with MIC values ranging from 32 to 128 μg/mL [67]. Two isoprenylisoindole alkaloids, diaporisoindoles A-B **100**–**101** (Figure 8), were obtained from the endophytic fungus *Diaporthe* sp. SYSU-HQ3, which was isolated from a fresh branch of the mangrove plant *Excoecaria agallocha*. Compound **100** showed potent inhibition activity against *Mycobacterium tuberculosis* protein-tyrosine phosphatase B, with an IC_50_ value of 4.2 µM [68].

In a nutshell, anti-angiogenic and anti-inflammatory activities were the main activities of alkaloids in both plants and endophytic fungi. In addition, phomaketides and their derivatives that were isolated from fungal endophytes possess antimicrobial activity just as those isolated in plants; therefore, alkaloids producing endophytic fungi can be used in the development of anti-angiogenic, anti-inflammatory, and antimicrobial drugs for both human and animal use.

### 2.3. Terpenoids

#### 2.3.1. Sesquiterpenoids and Their Derivatives 

**Figure 9:**
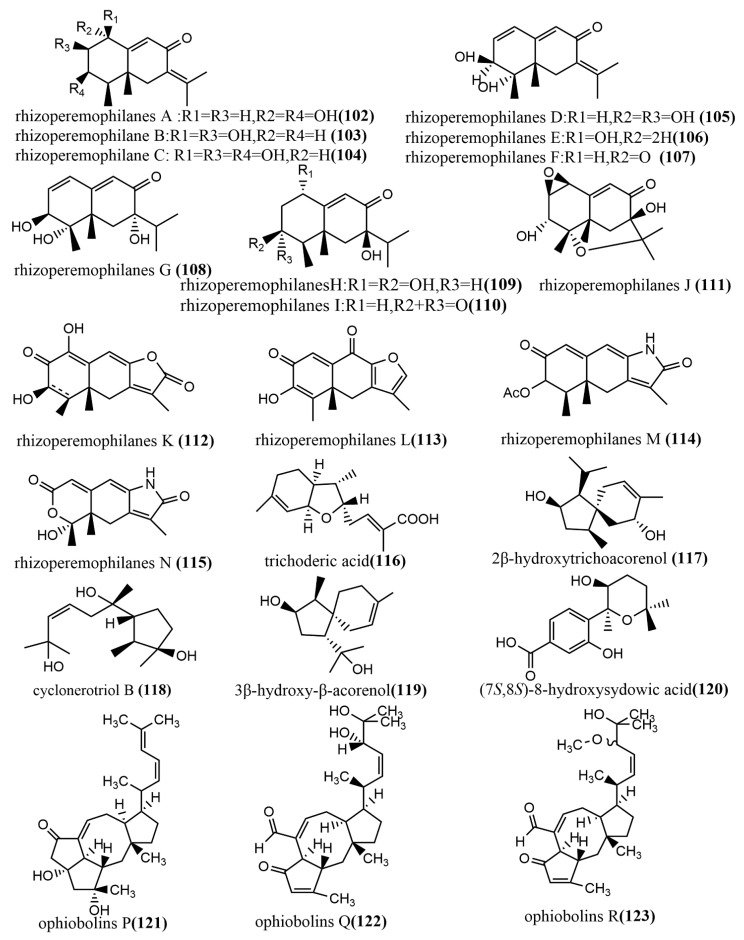

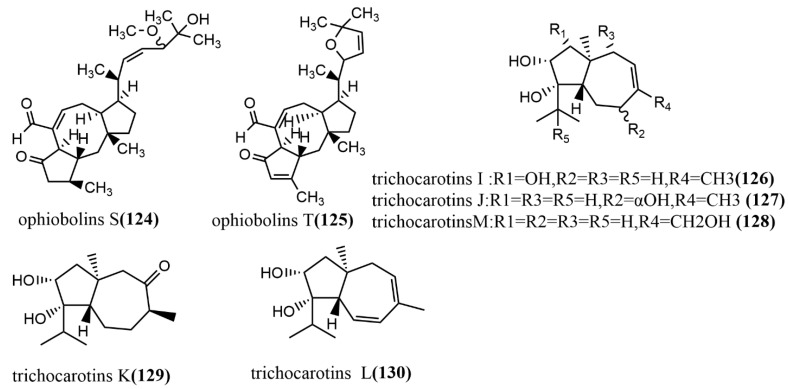
Chemical structures of sesquiterpenoids and derivatives.

The 1-methoxypestabacillin B **107** (Figure 9) was obtained from brown rice cultures of endophytic fungus *Diaporthe* sp. SCSIO 41011 isolated from the stem of the mangrove plant *Rhizophora stylosa*. Compound **107** was evaluated for the reversal of HIV incubation period and anti-influenza A virus activities, to which compound **107** did not show antiviral activity. However, its structure could serve as the backbone for the synthesis of more potent antiviral compounds [69]. The eremophilane-type sesquiterpenoids rhizoperemophilanes A-N **102**–**115** (Figure 9) were isolated from the ethyl acetate extract of *Rhizopycnis vagum* Nitaf22. Compound **111** contained a C-4/C-11 epoxide, and compound **115** had a 3-nor-eremophilane lactone-lactam skeleton. All compounds were evaluated for their cytotoxic activities against five tested human cancer cells (BGC823, Daoy, HCT116, HepG2, and NCI-H1650) and inhibition activities against radicle growth in rice seedlings. Compound **115** showed high selective cytotoxicity against NCI-H1650 and BGC823 cell lines, with IC_50_ values of 15.8 µM and 48.2 µM, respectively, while no significant cytotoxic activity was observed for other compounds at IC_50_ > 50 μm. Compounds **106**–**107** and **113**–**114** showed strong phytotoxic activities against radicle growth in rice seedlings at a concentration of 200 µg/mL, where the inhibition exceeded 50% [70]. The bisabolane-type sesquiterpene, trichoderic acid **116,** (Figure 9) and acorane-type sesquiterpene, 2β-hydroxytrichoacorenol **117** (Figure 9), were obtained from *Trichoderma* sp. PR-35 culture, an endophytic fungus isolated from stems of *Paeonia delavayi*. Compounds **116**–**117** were tested for antimicrobial activity against two pathogens (*Escherichia coli*, and *Shigella sonnei*) using an agar diffusion method. Compounds **116**–**117** showed moderate to weak antimicrobial activities, with MIA values ranging from 50 to 175 µg/mL [69]. The ring flores aurantii alkane-type sesquiterpene, cyclonerotriol B **118** (Figure 9), and the α-pinene skeleton-containing sesquiterpene, 3β-hydroxy-β-acorenol **119** (Figure 9), were obtained from *Fusarium proliferatum* AF-04 isolated from *Chlorophytum comosum* roots via a combination of high-performance liquid chromatography (HPLC) and a bioassay-guided method. Compounds **118**–**119** showed weak antimicrobial activities (MIC values > 100 μg/mL) against *Bacillus subtilis*, *Clostridium perfringens*, *E. coli*, and methicillin-resistant *Staphylococcus aureus* (MRSA) [71]. The aromatic bisabolene-type sesquiterpene (7S, 8S)-8-hydroxysydowic acid **120** (Figure 9) was obtained from the brown rice culture of the endophytic fungus *Aspergillus sydowii* EN-434 isolated from the marine red alga *Symphyocladia latiuscula* from Qingdao, China. Compound **120** showed potent DPPH radical scavenging activity, with an IC_50_ value of 113.5 μmol/L [72]. The ophiobolane sesquiterpenes ophiobolins P–T **121**–**125** (Figure 9) were isolated from the acetone extract of the endophytic fungus *Ulocladium* sp. using the one-strain many-compound (OSMAC) strategy. Compounds **121**–**125** were evaluated for their cytotoxicity and antibacterial activities against two tested human cancer cell lines (KB and HepG2 cell lines) and three tested pathogens (*Bacillus subtilis*, MRSA, and *Bacille* Calmette-Guerin). Compounds **121**–**125** showed moderate antimicrobial activities against *B. subtilis* and multi-drug-resistant *S. aureus*, with MIC values ranging from 15.6 to 62.5 μM. Compound **125** showed moderate antimicrobial activity against *Bacille* Calmette-Guerin, with an MIC value of 31.3 μM. Additionally, compound **125** showed potent cytotoxic activity against the HepG2 cell line, with an IC_50_ value of 0.24 μM, which was stronger than the positive control etoposide (IC_50_ value of 2.02 μM) [73]. The daucane-type sesquiterpenes trichocarotins I-M **126**–**130** (Figure 9) were obtained from *Trichoderma virens* QA-8 isolated from the roots of *Artemisia argyi* H. Lév. and Vaniot, and these compounds showed significant antimicrobial activities against *E. coli* EMBLC-1, with MIC values ranging from 0.5 to 16 μg/mL [74]. 

#### 2.3.2. Diterpenoids

**Figure 10:**
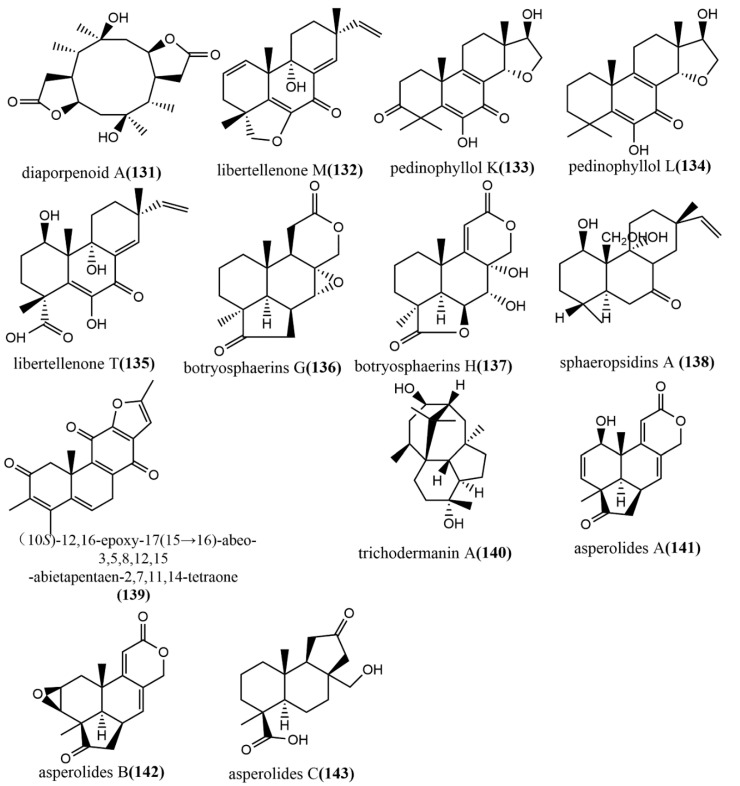
Chemical structures of diterpenoids and derivatives.

The ring diterpene diaporpenoid A **131** (Figure 10), containing a 5/10/5-fused tricyclic ring system, was isolated from the MeOH extract obtained from cultures of the mangrove endophytic fungus *Diaporthe* sp. QYM12. Compound **131** showed significant anti-inflammatory activity by inhibiting LPS-induced NO production in a mouse macrophage cell line RAW264.7, with an IC_50_ value of 21.5 μM [75]. The pimarane-type diterpene Libertellenone M **132** (Figure 10) was isolated from the marine source endophytic fungus *Phomopsis* sp. S12. Compound **132** inhibited pro-inflammatory cytokines IL1β and IL-18 mRNA expression in colon tissue, significantly reduced the cleavage of pro-caspase1, and dose-dependently inhibited the NF-κB nuclear translocation in macrophages. Clinical indications of acute colitis induced by 3% dextran sulphate sodium in mice were attenuated by intravenous administration of different doses of compound **132** (10 or 20 mg/kg), which is a potent inhibitor of NLRP3 inflammatory vesicles and may be a new medicine for treating acute colitis [76]. Three pimarane-type diterpenoids—pedinophyllol K **133** (Figure 10), pedinophyllol L **134** (Figure 10), and libertellenone T **135** (Figure 10)—were isolated from the endophytic fungal *Phomopsis* sp. S12 culture using the OSMAC strategy. The anti-inflammatory activities of all compounds were assessed using an LPS-induced inflammation model of mouse macrophage RAW264.7. Compound **135** dose-dependently inhibited the expression of inflammatory factors IL-1β and IL-6 at the mRNA level. Additionally, the anti-inflammatory activity of compounds **133**–**134** was similar to that of compound **135** in terms 0f IL-6 inhibition [77]. Two tetranorlabdane diterpenoids botryosphaerins G–H **136**–**137** (Figure 10) were obtained from the ethyl acetate extract of *Botryosphaeria* sp. P483 isolated from the branches of the herb *Huperzia serrata* (Thunb.) Trev. and tested for their antifungal activities against *Gaeumannomyces graminis*, *Fusarium solani*, and *Pyricularia oryzae* by the disk diffusion method. Compound **137** showed effective antifungal activity at a concentration of 100 μg/disk with an inhibitory zone diameter of 9 mm. (The inhibitory zone diameter of positive control carbendazim was 15–18 mm.) Compounds **136**–**137** were evaluated for their nematicidal activities against *Panagrellus redivivus* and *Caenorhabditis elegans* and showed weak nematicidal activities, with 30% and 28% fatality rates at a 24h action concentration of 400 mg/L, respectively [78]. The isopimarane diterpene sphaeropsidin A **138** (Figure 10) was isolated from the ethyl acetate extract of the endophytic fungus *Smardaea* sp. AZ0432 of *Ceratodon purpureus*. The in vitro cytotoxic activities of compound **138** against five human cancer cell lines (NCI- H460, MDA-MB-231, MCF-7, PC-3M, and SF-268) and human embryonic lung fibroblast cell line WI-38 were evaluated using the resazurin colorimetric assay. The results showed that compound **138** showed a high cell selectivity when it was applied at a concentration of 10 μM for 72 h and inhibited the migration of MDA-MB-231 cells by 50% at a subcytotoxic concentration of 1.5 μM [79]. (10S)-12,16-epoxy-17(15→16)-abeo-3,5,8,12,15-abietapentaene-2,7,11,14-tetraone **139** (Figure 10) was obtained from the cultures of the endophytic fungus *Pestalotiopsis adusta* isolated from stems of the medicinal plant *Clerodendrum canescens*. The cytotoxicity of compound **139** to the HL-60 tumor cell line was evaluated using the MTT assay, by which compound **139** showed moderate cytotoxic activity, with an IC_50_ value of 12.54 μM [80]. (The IC_50_ value of the positive control cisplatin was 9.20 μM.) The trichodermanin A **140** (Figure 10), a diterpene containing a 6-5-6-6 ring system, was obtained from the endophytic fungus *Trichoderma atroviride* S361 of *Cephalotaxus fortunei* and was not tested for any biological activities [81]. Therefore, further studies are needed to identify the potential biological activity of this compound in the future. The new tetranorlabdane diterpenoids, asperolides A–C **141**–**143** (Figure 10), were isolated from the ethyl acetate extract of the marine brown alga *Aspergillus wentii* EN-48 and the cytotoxic activities of compounds **141**–**143** to seven tested human cancer cell lines (NCI-H460, MDA-MB-231, HeLa, MCF-7, SMMC-7721, HepG2, and SW1990) were evaluated using the MTT method. Compounds **141**–**143** showed moderate cytotoxic activities, with IC_50_ values ≤ 10 Μm [82].

#### 2.3.3. Triterpenoids

**Figure 11:**
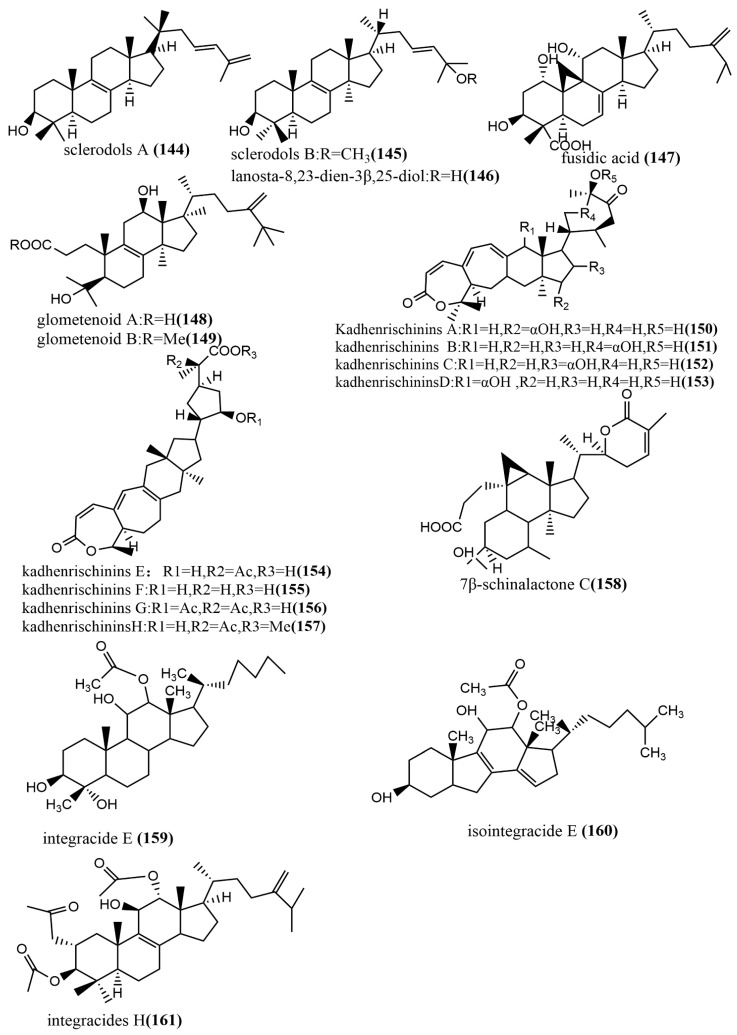

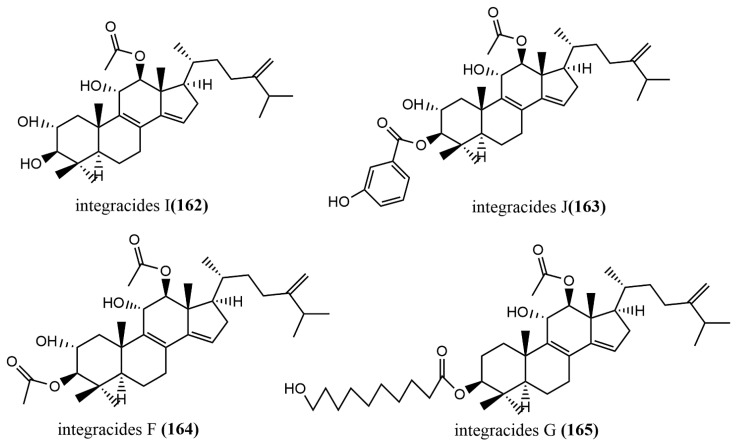
Chemical structures of terpenoids.

The 24-homo-30-nor-cycloartane triterpenoid **154** (Figure 11) was isolated from the endophytic fungus *Mycoleptodiscus indicus* FT1137. Compound **154** showed no activity against the human ovarian cancer cell line A2780 at a concentration of 20 μg/mL [83]. Three Lanostane-type triterpenes—sclerodols A–B **144**–**145** (Figure 11) and lanosta-8,23-dien-3β,25-diol **146** (Figure 11)—were obtained from *Eucalyptus grandis* cultures derived from the endophytic fungus *Scleroderma* UFSMSc1, and the antifungal activities of compounds **144**–**146** against *Candida albicans* and *Candida parapsolosis* were evaluated by the agar diffusion method. Compounds **144**–**146** showed moderate to weak antifungal activities, with MIC values ranging from 12.5 to 50 μg/mL. The antifungal effects of these compounds against *C. albicans* were associated with the inhibition of the selenocysteine methyltransferase (SMT) activity [84]. Fusidic acid **147** (Figure 11) was obtained from the cultures of the endophytic fungus *Acremonium pilosum* F47, isolated from the stem of *Mahonia fortunei* using the bioactivity-guided assay, and the antimicrobial activities of compound **147** against four human pathogens were tested (*S. aureus* ATCC 6538, *B. subtilis* ATCC 9372, *P. aeruginosa* ATCC 27853, and *E. coli* ATCC 25922) and evaluated. Compound **147** showed effective antimicrobial activities against *S. aureus* ATCC 6538 and *B. subtilis ATCC* 9372. The acetylation of the C-16 hydroxyl group of compound **147** was essential for antimicrobial action [85]. Two new ring *A*-cleaved lanostane-type triterpenoids, glometenoid A–B **148**–**149** (Figure 11), were obtained from the ethyl acetate extract of the mason pine endophytic fungus *Glomerella* sp. F00244. The cytotoxic activity of compounds **148**–**149** against the human ovarian cancer cell line HeLa was tested using the MTT assay. Compound **148** showed weak cytotoxic activity at a concentration of 10 μM with 21% inhibition [83]. Nine highly oxygenated schitriterpenoids—kadhenrischinins A–H **150**–**157** (Figure 11) and 7β-schinalactone C **158** (Figure 11)—were isolated from *Penicillium* sp. SWUKD4.1850, and compounds **154**–**157** contained a unique 3-one-2-oxabicyclo [1,2,3]-octane motif. All compounds were tested for their cytotoxic activities against the HepG2 tumor cell lines using the MTT assay, and these compounds showed weak cytotoxic activities, with IC_50_ values ranging from 14.3 to 40 μM [86]. Two tetracyclic triterpenoids—integracide E **159** (Figure 11) and isointegracide E **160** (Figure 11)—were isolated from the mycelia of *Hypoxylon* sp. 6269. Compound **159** showed weak inhibition activity against the HIV-1 integrase, with an IC_50_ value of 31.63 μM [87]. The tetracyclic triterpenoids, integracides H–J **161**–**163** (Figure 11), were obtained from the endophytic fungus *Fusarium* sp., which was isolated from the roots of *Mentha longifolia* L. (Labiatae) and were evaluated for antileishmanial activity against *L. donovani* promastigotes. Compound **161** showed significant antileishmanial activity, with an IC_50_ value of 4.75 μM, exceeding the positive control Pentamidine (IC_50_ value of 6.35 μM) [88]. The tetracyclic triterpenoids, integracides F–G **164**–**165** (Figure 11), were obtained from the endophytic fungus *Fusarium* sp. of *Mentha longifolia* L. (Labiatae). Compounds **164**–**165** were evaluated for their antileishmanial and cytotoxic activities to BT-549 and SKOV-3 cells and *Leishmania donovani* promastigotes. Compounds **164**–**165** showed significant cytotoxic activities against SKOV-3 and BT-549 cell lines, with IC_50_ values ranging from 0.16 to 1.97 μg/mL and 0.12 to 1.76 μg/mL, respectively. (The IC_50_ value of the positive control Pentamidine was 2.1 μg/mL.) Compounds **164**–**165** showed potent antileishmanial activities against *L. donovani* promastigotes, with IC_50_ values of 3.74 μg/mL and 2.53 μg/mL, respectively [89].

#### 2.3.4. Meroterpenoids

**Figure 12:**
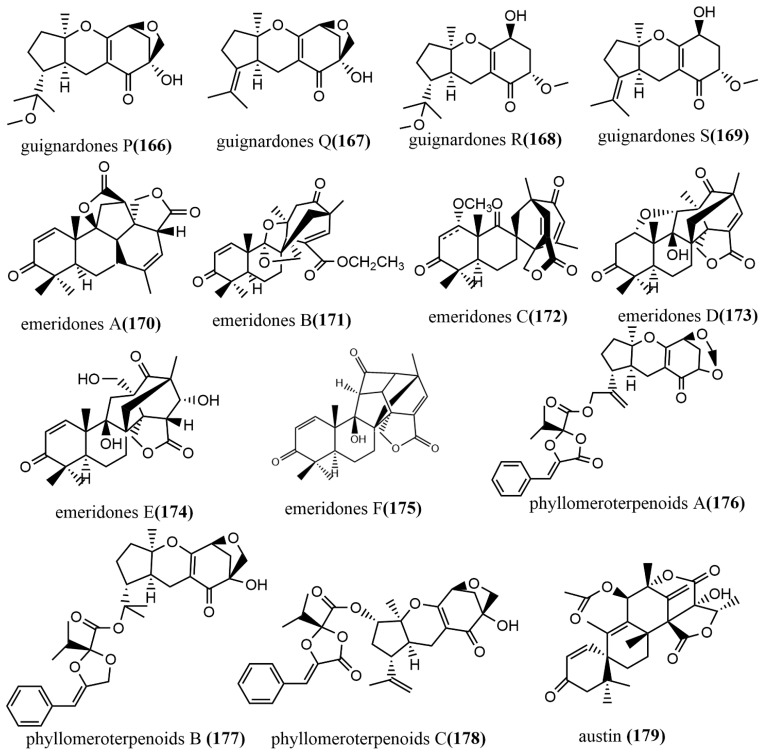
Chemical structures of Meroterpenoids.

Guignardones P–S **166**–**169** (Figure 12) were obtained from *Guignardia mangiferae* A348 cultures, and the cytotoxic activities of compounds **166**–**169** against three human cancer cell lines (SF-268, MCF-7, and NCI-H460) were tested using an MTT assay. Compounds **167** and **169** only showed weak cytotoxic activities against MCF-7 cell lines, with IC_50_ values ranging from 83.7 to 92.1 µM [90]. Six 3, 5-demethylorsellinic acid-based meroterpenoids emeridones A–F **170**–**175** (Figure 12) were isolated from *Emericella* sp. TJ29 cultures. Compound **171** possessed a 2,6 dioxabicyclo [2.2.1] heptane and a spiro [bicycle [3.2.2] nonane-2,1′-cyclohexane] moiety. The cytotoxic activities of all compounds against five human cancer cell lines (HL-60, SMMC7721, A549, MCF-7, and SW-480) were tested using the MTT assay, and compounds **172**, **173**, and **175** showed moderate cytotoxic activities against all tested cell lines, with IC_50_ values ranging from 8.19 to 18.8 µM [91]. Phyllomeroterpenoids A–C **176**–**178** (Figure 12) were isolated from the crude extract of *Phyllosticta* sp. J13-2-12Y fermentation broth. Compounds **176**–**178** showed moderate antimicrobial activities against *Staphylococcus aureus* 209P, *Candida aureus* 209P, and *Candida albicans* FIM709, with MIC values ranging from 32 to 128 μg/mL [92]. Austin **179** (Figure 12) was obtained from the ethyl acetate extract of *Talaromyces purpurogenus* H4 and *Phanerochaete* sp. H2 co-cultures, which showed moderate trypanocidal activity against *T. cruzi* at a concentration of 100 μg/mL, with an IC_50_ value of 36.6 µM. Notably, neither of the two endophytic fungi produced compound **179** when cultured separately under similar conditions [93].

To sum up, Meroterpenoids and their derivatives, which are mainly known for their antifungal properties in most plants species, have been found in endophytic fungi. However, recent studies have also reported anti-oxidative, anti-inflammatory, and anti-cancer activities from these compounds. Therefore, these microorganisms can be used in the development of drugs candidates for human, animal, and other agricultural activities.

### 2.4. Lactones

**Figure 13:**
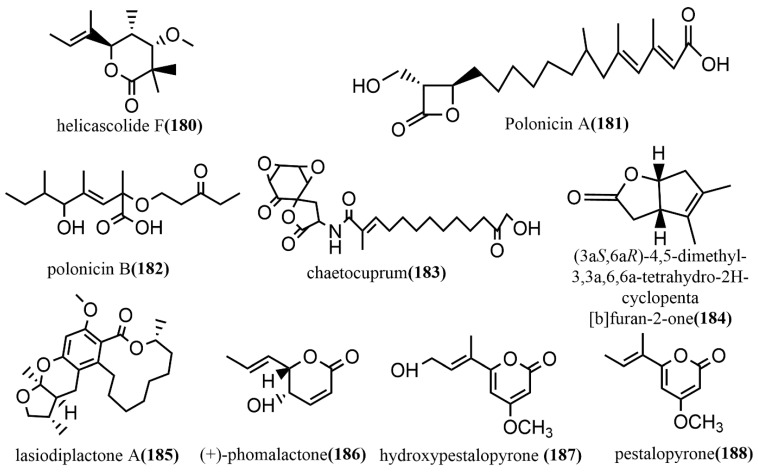
Chemical structures of Lactones.

Helicascolide F **180** (Figure 13) was obtained from *Talaromyces assiutensis* JTY2 isolated from *Ceriops tagal* leaves. The cytotoxic activities of compound **180** against three human cancer cell lines (HeLa, MCF-7, and A549) were tested using an MTT assay, in which compound **180** showed a moderate cytotoxic effect on all tested cell lines, with an IC_50_ value range of 14.1–38.6 μM [94]. Two β-lactones, polonicin A–B **181**–**182** (Figure 13), were obtained from the brown rice culture of the endophytic fungus *Penicillium polonicum* in the fruit of *Camptotheca acuminata*. Compound **181** showed effective glucose uptake activity at a concentration of 30 μg/mL on rat skeletal myoblast cell line L6, which enhanced 1.8-fold compared to that of the control. Compound **182** was used to assess its effect on GLUT4 translocation by using the fluorescent protein, IRAP-mOrange, which is stably expressed in L6 cells. It showed a 2.1-fold increase in fluorescence intensity on L6 cell membranes compared to the untreated controls [95]. The spirodilactone compound chaetocuprum **183** (Figure 13) was obtained from cultures of the endophytic fungus *Chaetomium cupreum* of wild *Anemopsis californica* from New Mexico, U.S.A. Compound **183** showed a weak antimicrobial activity against *S. aureus*, with an MIC value of 50 μg/mL [96]. A phytotoxic bicyclic lactone, (3aS,6aR)-4,5-dimethyl-3,3a,6,6a-tetrahydro-2H-cyclopenta [b] furan-2-one **184** (Figure 13), was obtained from the fermentation broth of *Xylaria curta* 92092022. Compound **184** contained a rare 5/5 rings-fusion system and was tested for antimicrobial activities against four pathogens (*Pseudomonas aeruginosa* ATCC 15442, *Staphylococcus aureus* NBRC 13276, *Aspergillus clavatus* F318a, and *Candida albicans* ATCC 2019) and the phytotoxicity against lettuce seedlings. Compound **184** showed moderate antimicrobial activities against *Pseudomonas aeruginosa* ATCC 15442 and *Staphylococcus aureus* NBRC 13276 at a concentration of 100 μg/disk, with inhibitory zone diameters of 13 mm and 12 mm, respectively. At the concentration of 25 μg mL ^−1^, compound **184** showed 50% inhibition on lettuce roots with a root length of 1.6 ± 0.3 cm (3.2 ± 0.5 cm for the control). At a concentration of 200 μg mL ^−1^, compound **184** strongly inhibited lettuce seed germination, with 90% inhibition [97]. Lasiodiplactone A **185** (Figure 13) was obtained from the mangrove endophytic fungus *Lasiodiplodia theobromae* ZJ-HQ1 and contained a unique tetracyclic system (12/6/6/5) of RAL 12 (12-membered β-resorcylic acid lactone) with a pyran ring and a furan ring. Compound **185** showed significant anti-inflammatory activity by inhibiting the LPS-induced NO production in RAW 264.7 cells, with an IC_50_ value of 23.5 μM, which was stronger than the positive control indomethacin (IC_50_ = 26.3 μM). Additionally, compound **185** showed potent α-glucosidase inhibition activity, with an IC_50_ value of 29.4 μM, which was superior to the commonly used clinical drug acarbose (IC_50_ = 36.7 μM) [98]. (+)-phomalactone **186** (Figure 13), hydroxypestalopyrone **187** (Figure 13), and pestalopyrone **188** (Figure 13) were isolated from the endophytic fungus *Aspergillus pseudonomiae* J1 cultures and evaluated for in vitro anti-trypanosomal activity against the *Trypanosoma cruzi* Y strain using an anti-epimastigote assay. Compounds **186**–**188** showed moderate to weak anti-trypanosomal activities, with IC_50_ values of 0.86 μM, 88.33 μM, and 580.19 μM, respectively [99].

In summary, this review reported that fungal endophytes could produce Lactones and their derivatives through their metabolic activities. In addition, these compounds possessed biological activities, such as antimicrobial, anti-cancer, allelopathic, and anti-inflammatory; thus, fungal endophytes that produce these compounds may be utilized in the pharmacological setup as alternatives to plant-derived compounds. 

### 2.5. Anthraquinones, Quinones, and Related Glycosides

**Figure 14:**
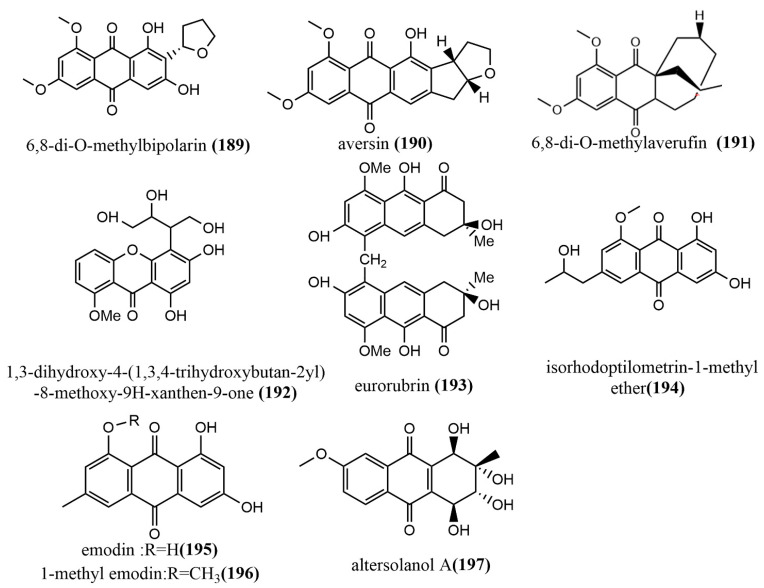
Chemical structure of anthraquinones, quinones, and related glycosides.

6,8-di-O-methylbipolarin **189** (Figure 14), aversin **190** (Figure 14), and 6,8-di-O-methylaverufin **191** (Figure 14) were obtained from rice cultures of the marine red algae endophytic fungus *Acremonium vitellinum* from Qingdao, China. Compounds **189**–**191** showed moderate insecticidal activities against the third-instar larvae of *Helicoverpa armigera*, with LC_50_ values of 0.72 mg/mL, 0.78 mg/mL, and 0.87 mg/mL, respectively. (The LC_50_ value for the positive control, matrine, was 0.29 mg/mL.) Additionally, the molecular mechanism of the insecticidal activity of compound **191** was investigated based on transcriptome sequencing. The identification of 5,732 differentially expressed genes was performed, of which 2,904 genes were downregulated and 2,828 genes were upregulated. The upregulated genes were primarily involved in cell autophagy, apoptosis, DNA mismatch repair, and replication [100]. A new quinone, identified as 1,3-dihydroxy-4-(1,3,4-trihydroxybutan-2-yl)-8-methoxy-9H-xanthen-9-one **192** (Figure 14), was obtained from *Phomopsis* sp. isolated from the rhizome of *Paris polyphyllavar.* in Yunnan, China. Compound **192** showed significant cytotoxic activities against A549 and PC3 cell lines, with IC_50_ values of 5.8 μM and 3.6 μM, respectively [101]. The anthraquinone derivative eurorubrin **193** (Figure 14) was obtained from the ethyl acetate extract of the endophytic fungus *Eurotium cristatum* EN-220 of the seaweed *Sargassum thunbergii* and tested for its antimicrobial activities against three tested pathogens (*E. coli*, *Physalospora obtuse*, and *Valsa mali)*, including its fatal activity against brine shrimp larvae. Compound **193** only showed a weak antimicrobial activity against *E. coli*, with an MIC value of 64 μg/mL. At the concentration of 10 μg/mL, compound **193** showed moderate fatal activity against brine shrimp larvae, with a fatality rate of 41.4% [102]. Isorhodoptilometrin-1-methyl ether **194** (Figure 14), emodin **195** (Figure 14), and 1-methyl emodin **196** (Figure 14) were obtained from cultures of the endophytic fungus *Aspergillus versicolor* of the red seaweed *Halimeda opuntia*. Compounds **194**–**196** were evaluated for their inhibiting activities against the hepatitis C virus NS3/4A protease, where Compounds **195**–**196** showed weak inhibition activities, with IC_50_ values ranging from 22.5 to 40.2 μg/mL [103]. The quinone altersolanol A **197** (Figure 14) was isolated from the endophytic fungus *Stemphylium globuliferum* of the medicinal plant *Mentha pulegium* (Lamiaceae). Compound **197** inhibited the proliferation of K562 and A549 cells in a time-dependent, dose-dependent manner and caused apoptosis by cleaving Caspase-3 and Caspase-9 and decreasing anti-apoptotic protein expression [104]. 

Anthraquinones, quinones, and related glycosides are known for their anti-viral and anti-apoptotic activity both in vitro and in vivo. Interestingly, these compounds have been identified and isolated from fungal endophytes by various studies and have similarly shown anti-viral and anti-apoptotic activities. Thus, endophytes that produce these compounds may serve as cheap and environmentally friendly alternative sources for the development of antimicrobial drugs instead to plant sources. 

### 2.6. Steroids

**Figure 15:**
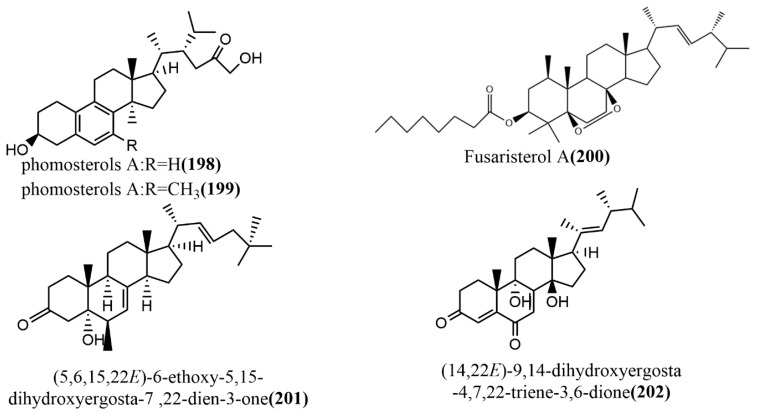
Chemical structures of steroids.

Phomosterols A–B **198**–**199** (Figure 15) were isolated from the endophytic fungus *Phoma* sp. SYSU-SK-7 of mangrove plants. Compounds **198**–**199** had an unusual aromatic B ring skeleton and showed significant inhibition activities against LPS-induced NO production in RAW 264.7 cells, with IC_50_ values of 13.5 μM and 25.0 μM, respectively. Additionally, compounds **198**–**199** showed potent α-glucosidase inhibition activities with IC_50_ values of 51.2 μM and 46.8 μM, respectively, exceeding the positive control 1-deoxynojirimycin (IC_50_ value of 62.8 μM) [105]. The ergosterol derivative fusaristerol A **200** (Figure 15) was obtained from the endophytic fungus *Fusarium* sp., which was isolated from the root of *Mentha longifolia* L. This compound showed significant antimicrobial activity against *Candida albicans*, with an MIC value of 8.3 μg/disc. Additionally, compound **200** showed moderate cytotoxic activity against human colorectal cancer cell line HCT 116, with an IC_50_ value of 0.21 μΜ, compared to the positive control adriamycin (IC_50_ value of 0.06 μΜ) [106]. (5,6,15,22E)-6-ethoxy-5,15-dihydroxyergosta-7,22-dien-3-one **201** (Figure 15) and (14,22E)-9,14-dihydroxyergosta-4,7,22-triene-3,6-dione **202** (Figure 15) were isolated from the endophytic fungus *Phomopsis* sp. of *Aconitum carmichaeli* in Yunnan, China. Compounds **201**–**202** were analyzed against six tested pathogenic fungi (*Candida albicans*, *Aspergillus niger*, *Fusarium avenaceum*, *Pyricularia oryzae*, *Hormodendrum compactum*, and *Trichophyton gypseum*) using a broth microdilution assay. Compounds **201**–**202** showed weak antifungal activities against *C. albicans* and *F. avenaceum*, with MIC values ranging from 64 to 128 μg/mL [107]. 

To summarize, endophytic fungi are alternative sources of steroids and their derivatives; thus, they may be harnessed for the production of various drugs since they have shown antimicrobial and anticancer activity in previous studies.

### 2.7. Other Types of Compounds

**Figure 16:**
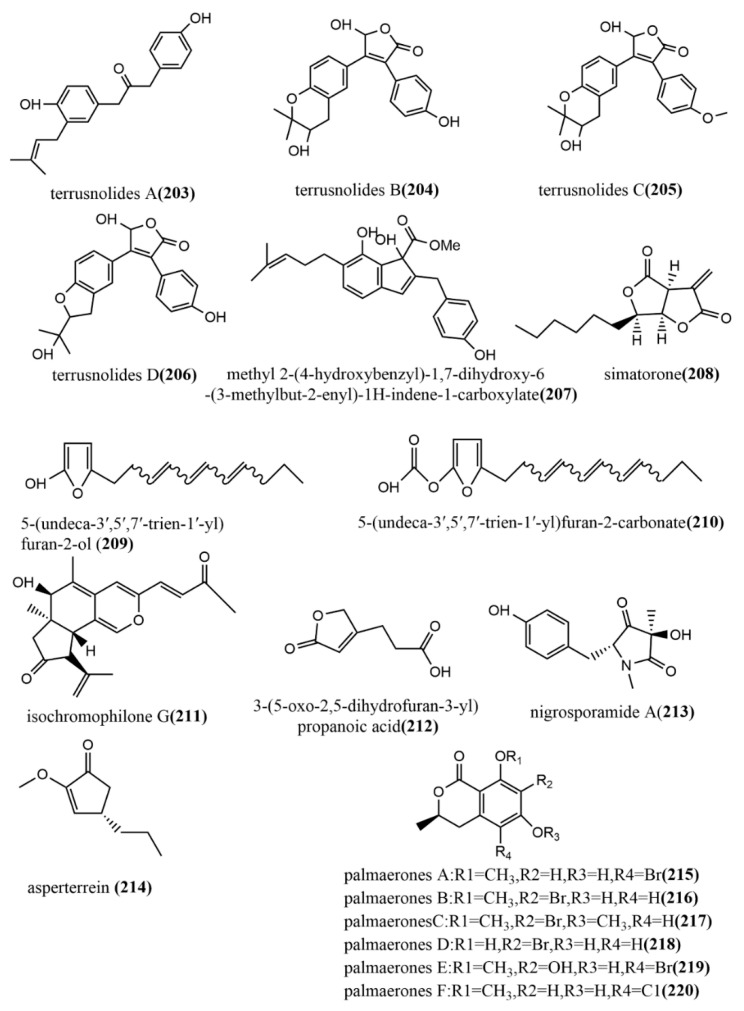
Chemical structures of other new compounds.

Four lignans, terrusnolides A–D **203**–**206** (Figure 16), were obtained from the endophytic fungus *Aspergillus* sp. isolated from the root of *Tripterygium wilfordii*. Compounds **203**–**206** showed significant inhibition of LPS-induced IL-1β, TNF-α, and NO production in RAW264.7 cells, with IC_50_ values ranging from 16.21 to 35.23 μΜ, 19.83 to 42.57 μΜ, and 16.78 to 38.15 μΜ, respectively, which were comparable to the positive control indomethacin (IC_50_ value of 15.67–21.34 μΜ) [108]. The indene derivative methyl 2-(4-hydroxybenzyl)-1,7-dihydroxy-6-(3-methylbut-2-enyl)-1H-indene-carboxylate **207** (Figure 16) obtained from the endophytic fungus *Aspergillus flavipes* Y-62 isolated from *Suaeda glauca* Bunge in Zhoushan, Zhejiang, China, showed weak antimicrobial activities against *Pseudomonas aeruginosa*, *Klebsiella pneumonia*, and *Staphylococcus aureus*, with MIC values ranging from 32 to 128 μg/mL [109]. The polychlorinated triphenyl diether simatorone **208** (Figure 16) was isolated from *Microsphaeropsis* sp. cultures, and its antimicrobial activities against three pathogens (*Escherichia coli*, *Bacillus megaterium*, and *Microbotryum violaceum*) were evaluated using an agar diffusion assay. Compound **208** showed effective antimicrobial activities against *B. megaterium* and *E. coli* with inhibitory zone diameters of 14 mm and 18 mm, respectively [110]. Two alkylated furan derivatives—5-(undeca-3′,5′,7′-trien-1′-yl) furan-2-ol **209** (Figure 16) and 5-(undeca-3′,5′,7′-trien-1′-yl) furan-2-carbonate **210** (Figure 16)—were obtained from the methanol extract of the endophytic fungus *Emericella* sp. XL029 isolated from *Panax notoginseng* leaves in Hebei, China. Compounds **209**–**210** both showed potent antifungal activities against six tested plant pathogenic fungi (*Rhizoctorzia solani*, *Verticillium dahliae Kleb*, *Helminthosporium maydis*, *Fusarium oxysporum*, *Fusarium tricinctum*, and *Botryosphaeria dothidea*), with MIC values ranging from 25 to 3.1 μg/mL [111]. The new azaphilone, isochromophilone G **211** (Figure 16), was obtained from the endophytic fungus *Diaporthe perseae* sp. isolated from *Pongamia pinnata* (L.) Pierre. Compound **211** showed significant DPPH and ABTS radical scavenging activities, with IC_50_ values of 7.3 μmol/mL and 1.6 μmol/mL, respectively [112]. The furan derivative, 3-(5-oxo-2,5-dihydrofuran-3-yl) propanoic acid **212** (Figure 16), was obtained from the endophytic fungus *Aspergillus tubingensis* DS37 isolated from *Decaisnea insignis* (Griff.) Hook & Thomson, and showed significant inhibition activities against *Fusarium graminearum* and *Streptococcus lactis*, with MIC values of 16 μg/mL and 32 μg/mL, respectively [113]. The pyrrolidinone derivative, nigrosporamide A **213** (Figure 16), was isolated from the endophytic fungus *Nigrospora sphaerica* ZMT05 of *Oxya chinensis* Thunberg and showed a three-fold higher α-glucosidase inhibition activity than the positive control acarbose (IC_50_ value of 446.7 µM) with an IC_50_ value of 120.3 µM. Compound **213** has the potential to be a lead compound for the development of α-glucosidase inhibitors [114]. The production of the terrein derivative asperterrein **214** (Figure 16) was induced by co-culturing endophytic fungi *Aspergillus terreus* EN-539 and *Paecilomyces lilacinus* EN-531 of the marine red alga *Laurencia okamurai*. Compound **214** showed weak antimicrobial activities against *Physalospora piricola* and *Staphylococcus aureus***,** with MIC values ranging from 32 to 64 μg/mL. Additionally, compound **214** was not detected in the sterile cultures of the two fungi alone [115]. The endophytic fungus *Lachnum palmae* of *Przewalskia tangutica* was isolated to halogenated dihydroisocoumarins palmaerones A–F **215**–**220** (Figure 16) under the guidance of UPLC-ESIMS. The antimicrobial activities of all compounds against five tested pathogens (*Cryptococcus neoformans*, *Penicillium sp.*, *Candida albicans*, *Bacillus subtilis*, and *Staphylococcus aureus*) were evaluated using the broth microdilution method. Compounds **215**–**220** showed potent to weak antimicrobial activities against all tested pathogens, with MIC values ranging from 10 to 55 μg/mL. Additionally, compounds **215** and **219** showed moderate inhibition of LPS-induced NO production in RAW264.7 macrophages, with IC_50_ values of 26.3 μM and 38.7 μM, respectively [116].

Over the past few years, plants have been a major source of numerous compounds that possess biological activities; however, this review revealed that most of these compounds were also produced by various endophytes, especially fungi. Therefore, the isolation and development of these compounds as novel drug candidates would be of great importance to the pharmacological industry since endophytes are easy to manage, keep, and work with compared with plants. Thus, we conclude that endophytic fungi may serve as alternative sources of bioactive compounds of pharmacological interest. 

All the information about the new compounds have been summarized below in Table 2.

## 3. Future Prospects and Challenges of Using Endophytic Fungi as an Alternative Source of Plant Bioactive Compounds 

Endophytic fungi are hidden and subtle dwellers in several plant tissues and intercellular spaces and can produce diverse chemical structures and efficient, low-toxic new secondary metabolites that were initially thought to be produced by the host plants. The current reports on the biosynthesis of plant metabolites by endophytic fungi, in conjunction with recent research advances in fermentation culture, extraction, isolation, and structure identification techniques, permit us to rapidly uncover new valuable compounds. Generally, fungi are chemically diverse, easily cultured, and biologically active modalities that have great flexibility to be regulated by adding precursors, elicitors, and specific enzymes to effectively increase the quantity and yield of bioactive compounds. Table 3 represents the culture conditions and specific bioactive secondary metabolites and yields produced by various endophytic fungi. Endophytic fungi can convert active compounds of the host plant into more potent derivatives. This makes endophytic fungi an alternative and sustainable source of plant bioactive compounds [117,118]. The search for new compounds in endophytic fungi requires specific theories and ingenious bioprospecting strategies. Along with the continuously growing literature reports, the most promising host plants can be selected. It includes the selection of (A) plants from special habitats or growing in biodiversity-rich areas, including mangrove plants in tropical marine intertidal zones, and (B) medicinal and indigenous plants with ethnopharmacological uses, including *Camptotheca acuminata* and *Ageratina adenophora*. These selection criteria provide a reference for the current and future screening of host plants for endophytic fungi with new bioactive compounds [119,120]. This review has summarized 220 new compounds obtained between 2011 and 2021 from endophytic fungi using different culture methods, including the common culture, co-culture with bacteria or other fungi, and the addition of metal ions. These new compounds have unique molecular structures, and these rare structures allow these compounds to possess diverse biological activities, including significant antimicrobial and cytotoxic activities and α-glucosidase inhibition. These compounds have the potential to be modified as pro-drug molecules or directly developed as drugs for treating certain diseases. However, most of the current studies on the activity of new compounds with endophytic fungal sources are limited to in vitro studies; therefore, animal experiments and human intervention clinical trials are needed to further investigate the in vivo activities and mechanisms of action of the new compounds. 

Unfortunately, endophytic fungi as new sources of bioactive secondary metabolites encounter various limitations, including the attenuated yield of secondary metabolites due to long-term storage and repeated passages under laboratory culture conditions, silencing of biosynthetic gene clusters or low level of expression (activation of gene clusters depends on environmental factors). Thus, the ability of endophytic fungi to produce new compounds of interest has been underestimated [129]. The expression could be upregulated by physicochemical and genetic manipulation techniques to increase the production of specific metabolites in endophytic fungi and to produce analogs of new active secondary metabolites. Methods including the OSMAC strategy (activation of silent biosynthetic gene clusters mediated by changes in medium composition, temperature, and aeration efficiency to produce desired metabolites), co-culture (mimicking natural ecosystems and triggering silent gene clusters to promote metabolite secretion and enhance bioactive metabolite production by microbial interaction-induced stress responses), and chemical epigenetic modification methods have been used to isolate new compounds. It was found that the addition of micromolar or even nanomolar small-molecule chemicals to cultures inhibits or activates relevant enzymes and remodels the fungal epigenome to increase the diversity of its secondary metabolites, including DNA methyltransferases (DNMTs) and histone deacetylase inhibitors (HDACs) [130,131]. The addition of epigenetic modifiers (5 μM SAHA and 10 μM AZA) to the endophytic fungus *Xylaria psidii* isolated from leaves of *Vitis vinifera* showed elevated resveratrol concentrations of 52.32 μg/mL and 48.94 μg/mL, respectively, by HPLC analysis (control concentration was 35.43 μg/mL). The treatments with 5 μM SAHA and 10 μM AZA showed stronger antioxidant activity with 30.92% and 33.82% DPPH radical scavenging, respectively, compared to the wild strain (19.26%) [132]. Unlike the chemical epigenetic modification methods reported, introducing exogenous substances as precursors into the cultures, including methyl jasmonate, causes the production of new compounds containing their structural units [133]. However, the addition of host plant components to the culture to induce the production of new compounds has rarely been reported. Additionally, it is necessary to elucidate the pathways by which endophytic fungi biosynthesize secondary metabolites, including the enzymes and genes involved via “omics” techniques—genomics, transcriptomics, and metabolomics—in regulating and manipulating the biosynthetic process to increase the number of new compounds [134]. 

## 4. Conclusions

Pharmaceutical chemists are turning their focus on the development of safe, efficient, and low-toxic new drugs from natural sources. Endophytic fungi may serve as renewable sources of novel bioactive compounds with pharmacological activities, as the number of new compounds to be isolated in the future tends to increase exponentially and rapidly. In addition, numerous studies have also reported that these bioactive compounds isolated from the endophytic fungi are also present in plants and have similar biological activities as the compounds from plant sources. Therefore, we conclude that endophytic fungi may be the best alternative for harnessing pharmacological bioactive compounds for the development of drugs for both human and animal use. Hence, there is a need for the identification of more compounds with pharmacological activity from endophytic fungi and elucidate their mechanisms of action through biological, pharmacodynamic, biochemical, bioinformatics, and pre-clinical approaches.

## Figures and Tables

**Table 1:** Several endophytic fungi of host plants have been reported to produce compounds with similar activity.

No.	Endophytic Fungus	Host Plant	Regions/Countries	Compound	Biological Activity	Ref.
1	*Lophiostoma* sp.	*Eucalyptus exserta*	Guangzhou, China.	Scorpinone	Antibacterial	[22]
2	*Mycosphaerella* sp.	*Myrciaria floribunda*	Amazon rainforest, Brazil.	Myriocin	Antifungal	[23]
3	*Mucor* sp.	*Centaurea stoebe*	Idaho, USA	Terezine E	Antifungal and cytotoxicity	[24]
4	*Aspergillus calidoustus*	*Acanthospermum australe*	Jalapao State Park, Tocantins, Brazil.	Ophiobolin K 6-epi-ophiobolin K	Antifungal, trypanocidal and cytotoxicity	[25]
5	*Phomopsis* sp.	*Garcinia kola (Heckel) nut*	Yaounde, Cameroon	Cytochalasins H	Antibacterial and cytotoxicity	[26]
6	*Aspergillus nidulans*	*Nyctanthes arbor-tristis Linn*	Karachi, Pakistan	Sterigmatocystin	Antiproliferative activity	[27]
7	*Trichoderma asperellum* and *Trichoderma brevicompactum*	*Vinca herbacea*	Hamedan, Iran	4b-hydroxy-12,13-epoxytrichothec-9-ene	Antimicrobial and antiproliferative activity	[28]
8	*Phyllosticta elongata*	*Cipadessa baccifera*	Western Ghats, India	Camptothecin	Anticancer agent	[29]
9	*Fusarium verticillioides*	*Huperzia serrata*	Gucheng Mountain, Sichuan, China	Huperzine A	Treatment of Alzheimer’s disease	[30]
10	*Fusarium solani*	*Cassia alata*	Bangladesh	Napthaquinones Azaanthraquinones	Cytotoxicity, antimicrobial and antioxidant activity	[31]
11	*Fusarium* sp. and *Lasiodiplodia theobromae*	*Avicennia lanata*	Terengganu, Malaysia	Anhydrofusarubin dihydrojavanicin	Antitrypanosomal	[32]
12	*Corynespora cassiicola*	*Gongronema latifolium*	Nigeria	Corynesidone D	Anti-inflammatory/anticancer agent	[33]
13	*Pestalotiopsis theae*	*Camellia sinensis Theaceae*	Hangzhou, China	punctaporonin H	Antibacterial and cytotoxicity	[34]
14	*Phialocephala fortinii*	*Podophyllum peltatum*	Tamilnadu, India	Podophyllotoxin	Antiviral, antioxidant, and antirheumatic activities	[35]

**Table 2:** Brief summary of new compounds.

Compound	Molecular Formula	Color and Morphology	Endophytic Fungus	Host Plant	Site and Nation	Pharmacological Activity	Ref.
Polyketides Chromones							
**1**	C_12_H_13_O_6_	colorless powder	*Botryosphaeria ramosa* L29	leaf of *Myoporum bontioides*	Leizhou Peninsula, China	Displayed acceptable antimicrobial activities against *Fusarium oxysporum*	[36]
**2**	C_14_H_15_O_6_	white powder					
**3**	C_11_H_11_O_5_						
**4**	C_17_H_19_N_3_O_3_S_2_	yellow crystals	*Phaeosphaeria fuckelii*	*Phlomis umbrosa*	Mount Hua, China	Mushroom tyrosinase inhibitory activity	[37]
**5**	C_15_H_16_O_7_S	yellow powder	*Chaetomium seminudum*			Showed antifungal activity (5–6); Exhibited radical scavenging activity against DPPH; Showed significant antioxidant activity ((5)	[38]
**6**	C_16_H_18_O_7_S						
**7**	C_16_H_24_O_5_	colorless oil	*Pestalotiopsis fici* W106-1	*Camellia sinensis*	Hangzhou, China	Displayed inhibitory effects on HIV-1 replication in C8166 cells ((7–8); Showed low to moderate cytotoxic activity (9–10); Displayed significant antifungal activity (9)	[39]
**8**							
**9**	C_32_H_54_O_6_						
**10**	C_32_H_54_O_6_Na						
Polyketides α-pyrones							
**11**	C_11_H1_4_O_4_	colorless crystals	*Neurospora udagawae*	shoot of *Quercus macranthera*		Exhibited moderate antifungal (vs. *Rhodoturula glutinis*) activity and cytotoxicity against KB3.1 cells (12)	[40]
**12**	C_10_H_10_O_4_	colorless oil					
**13**	C_28_H_42_O_4_	colorless amorphous powder	*Aspergillus niger* MA-132	*Avicennia marina*	Hainan Province, China	Showed potent antifungal and cytotoxic activities	[41]
**14**							
**15**	C_14_H_22_O_5_	yellow oil	*Pestalotiopsis fici*	branches of *Camellia sinensis* (Theaceae)	Hangzhou, China	Displayed significant antifungal against *Gibberella zeae*	[42]
	
**17**	C_10_H_14_O_4_	yellow oil	*Aspergillus oryzae*	*Paris polyphylla* var. *yunnanensis*	Dali, Yunnan Province, China	The biological activities of compounds 17–18 were not tested	[43]
**18**							
**19**	C_11_H_16_O_4_	yellow gum	*Penicillium herque*	*Cordyceps sinensis*	Xiahe, China	Weak cytotoxic activity	[44]
**20**	C_12_H_16_O_5_						
**21**							
Polyketides: Other polyketides							
**22**	C_22_H_35_ClO_7_	white powder	*Phoma* sp. NTOU4195	*Pterocladiella capillacea*	Taiwan, China	Showed potent anti-angiogenic activity (22); Exhibited inhibition of nitric oxide production in lipopolysaccharide (LPS)-stimulated RAW264.7 macrophage cells (24)	[45]
**23**							
**24**							
**25**							
**26**							
**27**	C_22_H_34_O_6_						
**28**	C_22_H_26_NO_8_						
**29**	C_24_H_35_NO_5_	colorless oil	*Simplicillium subtropicum* SPC3	fresh bark of *Duguetia* *staudtii*	Cameroon	Weak cytotoxic activity	[46]
**30**	C_24_H_35_NO_6_						
**31**	C_12_H_20_O_3_	colorless oil	*Cladosporium cladosporioides* MA-299	leaves of the mangrove plant *Bruguiera gymnorrhiza*	Hainan Island, China	Showed potent antimicrobial((vs. *Escherichia coli* and *Staphylococcus aureus*) activity and moderate inhibition activity against acetylcholinesterase (33)	[47]
**32**							
**33**	C_12_H_22_O_4_	pale yellow powder					
**34**	C_14_H_24_O_5_	pale yellow oil					
**35**	C_12_H_20_O_4_	colorless crystals					
**36**	C_14_H_20_O_5_	colorless powder	*Aspergillus fumigatiaffnis*	*Tribulus terestris*		Weak antimicrobial activities	[48]
**37**	C_14_H_12_O_6_Na	white amorphous solid	*Alternariaalternata* ZHJG5	leaf of *Cercis hinensis*	Nanjing, China	Exhibited potent antimicrobial activity; Showed significant protective effect against the bacterial blight of rice (37)	[49]
**38**	C_14_H_12_O_5_Na	white powder					
**39**	C_14_H_12_O_5_Na						
**40**	C_29_H_22_O_12_Na						
**41**	C_24_H_27_NO_5_	brown solid	*Peyronellaea* sp. FT431	healthy leaf of a Hawaiian indigenous plant, *Verbena* sp.	Lyon, France	Showed weak to moderate cytotoxic activity (41–42)	[50]
**42**	C_24_H_26_O_7_						
**43**	C_24_H_26_O_7_						
**44**	C_18_H_20_O_5_						
**45**	C_29_H_22_O_9_	red wine colored lump crystal	*Alternaria* sp. MG1	*Vitis quinquangularis*		Showed weak cytotoxicity	[51]
Alkaloids Cytochalasin							
**46**	C_22_H_32_N_2_O_5_	white amorphous solid	*Phomopsis* sp. sh917	Fresh stems of *I. eriocalyx* var. *laxiflora*	Kunming, China	Significant inhibitory activity against NO production in LPS-induced RAW264.7 cells (46)	[52]
**47**	C_22_H_33_NO_4_						
**48**							
**49**	C_32_H_40_N_2_O_6_	colorless amorphous powder	*Chaetomium globosum* TW1-1	*Armadillidium vulgare*	Hubei Province, China	Showed potential cytotoxic activities against cancer cell lines (HL-60, A-549, SMMC-7721, MCF-7, and SW-480)	[53]
**50**	C_32_H_38_N_2_O_6_						
**51**							
**52**	C_32_H_40_N_2_O_6_						
**53**	C_32_H_38_N_2_O_5_	white amorphous powder					
**54**	C_32_H_83_N_2_O_6_						
**55**	C_32_H_36_N_2_O_4_						
**56**		colorless amorphous powder					
**57**	C_34_H_42_N_2_O_7_Na						
**58**	C_28_H_37_NO_3_	white amorphous solid	*Diaporthe* sp. SC-J0138	*Cyclosorus parasiticus* (Thelypteridaceae)	Guangdong Province, China	Showed significant cytotoxic activities against four human cancer cell lines (A549, HeLa, HepG2, and MCF-7) (58); Exhibited selective cytotoxic activity (59–62)	[54]
**59**	C_28_H_37_NO						
**60**							
**61**	C_28_H_37_NO_4_						
**62**							
**63**	C_25_H_37_NO_4_	colorless crystal	*Cytospora chrysosperma* HYQZ-931	*Hippophae rhamnoides*		Exhibited significant antibacterial activity (63,65)	[55]
**64**		white amorphous powder					
**65**	C_26_H_41_NO_5_						
Alkaloids Indole alkaloids							
**66**	C_26_H_28_N_3_O_4_	brilliant yellowish powder	*Aspergillus* sp. YJ191021		Zhejiang Province, China	Exhibited moderate antibacterial activity (66); Displayed notable anti-inflammatory; Exhibited notable cytotoxicity (66–69)	[56]
**67**	C_26_H_29_N_3_O_5_	white powder					
**68**	C_27_H_31_N_3_O_6_Na						
**69**	C_27_H_31_N_3_O_6_						
**70**	C_28_H_31_N_3_O_6_						
**71**	C_26_H_31_N_3_O_6_						
**72**	C_30_H_33_NO_7_	white amorphous powder	*Penicillium chrysogenum* XNM-12	*Leathesia nana*	Shandong Province, China	Exhibited moderate antibacterial effects against *Ralstonia solanacearum*	[57]
**73**	C_23_H_38_N_1_NaO_3_	amorphous powder	*Hypomontagnella monticulosa* Zg15SU	fresh rhizome of *Zingiber griffithii* Baker	Indonesia	Showed potent cytotoxic activity	[58]
**74**	C_20_H_22_N_2_NaO_4_	yellowish powder	*Aspergillus lentulus* DTO 327G5	*Caenagrion*	Shanghai, China	Displayed weak to moderate antibacterial activity	[59]
**75**	C_19_H_21_O_4_N_2_	white powder					
**76**	C_24_H_25_N_3_NaO_3_						
Alkaloids Diketopiperazine derivatives							
**77**	C_20_H_27_N_3_O_3_S_2_Na	white solid powder	*Phaeosphaeria fuckelii*	*Phlomis umbrosa*	Mount Hua, China	Showed strong inhibitory effects on mushroom tyrosinase	[60]
**78**	C_26_H_29_N_3_O_5_	colorless oil	*Paecilomyces variotii* EN-291	*Grateloupia turuturu*	Qingdao Province, China	Exhibited potent antifungal effects	[61]
**79**	C_22_H_23_N_3_O_4_						
**80**	C_21_H_25_O_3_N_3_	yellow powder	*Aspergillus* sp. 16-5c	leaf of *S. apetala*	Hainan Island, China	Showed potent to moderate α-glucosidase inhibitory activity (80–81)	[62]
**81**	C_21_H_23_O_4_N_3_	white powder					
**82**	C_21_H_23_O_3_N_3_	yellow powder					
**83**	C_21_H_25_O_3_N_3_						
**84**	C_22_H_27_O_3_N_3_						
**85**	C_18_H_15_O_4_N_3_	white powder					
**86**	C_19_H_24_N_2_O_6_S	colorless crystals	*Penicillium brocae* MA-231	*Avicennia marina*		Displayed moderate to high activities against *Staphylococcus aureus*	[63]
**87**	C_19_H_22_N_2_O_5_S	yellowish solid					
**88**	C_20_H_26_N_2_O_6_S_2_	colorless crystals					
**89**	C_20_H_26_N_2_O_6_S_2_	colorless solid					
**90**	C_20_H_24_N_2_O_6_S_2_						
**91**	C_19_H_18_N_2_O_4_S	colorless crystals	*Penicillium brocae* MA-231			Showed moderate antimicrobial activities against *S. aureus* and *Aeromonas hydrophilia*	[64]
**92**	C_19_H_16_N_2_O_4_S						
**93**	C_18_H_14_N_2_O_4_						
Alkaloids: Other types of alkaloids							
**94**	C_21_H_16_N_4_O_2_	colorless needles	*Penicillium vinaceum* (X17)	corm of *Crocus sativus*	Shanghai, China	Showed weak cytotoxic activities against three human tumor cell lines (A549, LOVO, and MCF-7)	[65]
**95**	C_14_H_16_Br_2_N_2_O_5_	colorless amorphous powder	*Acanthodendrilla* sp.		Thailand	Exhibited efficient and selective cytotoxic activities against two human tumor cell lines (H292 and HaCaT)	[66]
**96**							
**97**	C_19_H_20_NO3	colorless needles crystal	*Penicillium sumatrense* GZWMJZ-313	leaf of *Garcinia multiflora*	Guizhou, China	Showed moderate to weak antimicrobial activities against *Staphylococcus aureus*, *Pseudomonas aeruginosa*, and *Escherichia coli*	[67]
**98**	C_20_H_24_NO_3_	white powder					
**99**	C_20_H_21_NO_3_						
**100**	C_25_H_29_O_5_N	white powder	*Diaporthe* sp. SYSUHQ3	fresh branch of the mangrove plant *Excoecaria agallocha*		Showed potent inhibition activity against *Mycobacterium tuberculosis* protein-tyrosine phosphatase B	[68]
**101**	C_25_H_29_O_5_N						
Terpenoids Sesquiterpenoids and their derivatives							
**102**	C_15_H_22_O_3_Na	colorless oil	*Rhizopycnis vagum Nitaf22*	*Nicotiana tabacum*		Exhibited high selective cytotoxicity against NCI-H1650 and BGC823 cell lines (115); Showed strong phytotoxic activities against radicle growth in rice seedlings (106–107, 113–114)	[70]
**103**	C_15_H_23_O_3_						
**104**	C_15_H_22_O_4_Na	colorless amorphous solid					
**105**	C_15_H_20_O_3_Na	colorless oil					
**106**	C_15_H_24_O_3_Na						
**107**	C_15_H_22_NaO_3_						
**108**	C_15_H_21_O_4_						
**109**	C_15_H_25_O_4_						
**110**	C_15_H_22_O_3_Na						
**111**	C_15_H_19_O_5_	colorless amorphous solid					
**112**	C_15_H_13_O_5_	brown amorphous solid	*Rhizopycnis vagum Nitaf22* *Rhizopycnis vagum Nitaf22*	*Nicotiana tabacum* *Nicotiana tabacum*		Exhibited high selective cytotoxicity against NCI-H1650 and BGC823 cell lines (115); Showed strong phytotoxic activities against radicle growth in rice seedlings (106–107, 113–114)	[70]
**113**	C_15_H_14_O_4_Na	yellowish oil					
**114**	C_17_H_18_NO_4_	greenish-yellow amorphous solid					
**115**	C_14_H_15_NO_4_	light-yellowish amorphous solid					
**116**	C_15_H_22_O_3_	colorless oil	*Trichoderma* sp. PR-35	healthy stem of *Paeonia delavayi*	Yunnan Province, China	Showed moderate to weak antimicrobial activities against *Escherichia coli* and *Shigella sonnei*	[69]
**117**	C_15_H_26_O_2_						
**118**	C_15_H_28_O_3_Na	colorless oil	*Fusarium proliferatum* AF-04		Lanzhou, China	Displayed weak antimicrobial against *Bacillus subtilis*, *Clostridium perfringens*, *E. coli*, and MRSA	[71]
**119**	C_15_H_26_O_2_Na						
**120**	C_15_ H _20_ O_5_	colorless oil	*Aspergillus sydowii* EN-434	*Symphyocladia* *latiuscula*	Qingdao Province, China	Exhibited radical scavenging activity against DPPH	[72]
**121**	C_25_H_37_O_4_	amorphous powder	*Ulocladium* sp.		Yunnan Province, China	Showed moderate antimicrobial activities against *B. subtilis* and multi-drug-resistant *S. aureus* (121–125); Exhibited high selective cytotoxicity against the HepG2 cell line (125)	[73]
**122**							
**123**	C_26_H_38_O_4_Na						
**124**	C_26_H_40_O_5_Na						
**125**	C_25_H_34_O_3_Na						
**126**	C_15_H_26_O_3_	colorless crystals	*Trichoderma virens* QA-8	fresh inner root tissue of the grown medicinal herb *Artemisia argyi* H. Lév. and Vaniot	Hubei Province, China	Showed significant antimicrobial activities against *E. coli*	[74]
**127**		amorphous powder					
**128**		colorless oil					
**129**	C_15_H_24_O_2_						
**130**	C_15_H_26_O_3_	colorless waxy solid					
Terpenoids Diterpenoids							
**131**	C_20_H_32_O_6_Na	colorless oil	*Diaporthe* sp. QYM12	healthy leaves of *Kandelia candel*	Hainan Province, China	Showed significant anti-inflammatory effects through the inhibition of NO production	[75]
**132**	C_21_H_28_O_6_	colorless crystals	*Phomopsis* sp. S12	seed of *Illigera rhodantha*		Showed excellent inhibitory effects on the production of IL-1β and IL-18; Effects on the NF-κB signaling pathway	[76]
**133**	C_20_H_26_O_5_	colorless needle crystal	*Phomopsis* sp. S12	seed of *Illigera rhodantha*		Exhibited anti-inflammatory activity against the production of IL-1b and IL-6 induced by lipopolysaccharide (LPS) in macrophages	[77]
**134**	C_20_H_28_O_4_	colorless oil					
**135**	C_20_H_26_O_6_						
**136**	C_16_H_20_O_5_	colorless needles	*Botryosphaeria* sp. P483	Chinese Herbal Medicine *Huperzia serrata*	Kunming, China	Showed effective antifungal antifungal activities against *Gaeumannomyces graminis*, *Fusarium solani*, and *Pyricularia oryza* (136); Showed weak nematicidal activities	[78]
**137**	C_16_H_20_O_6_	colorless solid					
**138**	C_20_H_28_O_6_	white amorphous solid	*Smardaea* sp. AZ0432	photosynthetic tissue of the moss *Ceratodon purpureus*	Chiricahua Mountains of southeastern Arizona, USA	Exhibited selective cytotoxicity	[79]
**139**	C_20_H_16_O_5_	yellowish needles	*Pestalotiopsis* *adusta*	Fresh, healthy stems of *Clerodendrum canescens*	Yandang, Zhejiang Province, China	Demonstrated cytotoxic activities against the HL-60 tumor cell line	[80]
**140**	C_20_H_34_O_2_	colorless needles	*Trichoderma atroviride* S361	*Cephalotaxus fortunei*	Jiande, Zhejiang, China	Bioactivity tests were not performed	[81]
**141**	C_16_H_16_O_5_	colorless needles	*Aspergillus wentii* EN-48	unidentified marine brown algal species of the genus *Sargassum*	Qingdao Province, China	Showed moderate cytotoxic activities against seven human tumor cell lines (NCI-H460, MDA-MB-231, HeLa, MCF-7, SMMC-7721, HepG2, and SW1990)	[82]
**142**	C_16_H_16_O_5_						
**143**	C_16_H_24_O_5_						
Terpenoids Triterpenoids							
**144**	C_30_H_48_O	colorless solid	*Scleroderma* UFSMSc1	*Eucalyptus grandis*		Showed moderate to weak antifungal activities against *Candida albicans* and *Candida parapsolosis*	[84]
**145**							
**146**							
**147**	C_29_H_46_O_5_	white powder	*Acremonium pilosum* F47	pedicel of the Chinese medicinal plant *Mahonia fortunei*	Qingdao Province, China	Displayed effective antimicrobial activities against *S. aureus* and *B. subtili*	[85]
**148**	C_30_H_50_O_6_	yellow amorphous powder	*Glomerella* sp. F00244	stem of mason pine	Fujian Province, China	Showed weak cytotoxic activity (148)	[83]
**149**	C_31_H_52_O_6_	white amorphous powder					
**150**	C_30_H_40_O_6_	yellowish needle crystals	*Penicillium* sp. SWUKD4.1850	healthy branches of *Kadsura angustifolia*	Yunnan Province, China	Exhibited moderate in vitro cytotoxic activities	[86]
**151**	C_30_H_40_O_6_	white needle crystals					
**152**	C_30_H_40_O_6_	white amorphous solid					
**153**	C_30_H_41_O_6_						
**154**	C_32_H_44_O_7_	white amorphous powder					
**155**	C_30_H_42_O_6_	white powder					
**156**	C_34_H_46_O_8_	yellow amorphous solid					
**157**	C_31_H_44_O_6_						
**158**	C_30_H_46_O_6_	white amorphous powder					
**159**	C_32_H_50_O_5_	white amorphous powder	*Hypoxylon* sp. 6269	*Artemisia annua*		Weak inhibition activity against the HIV-1 integrase (159)	[87]
**160**	C_29_H_44_O_4_						
**161**	C_36_H_55_O_7_	white amorphous powder	*Fusarium* sp.	roots of *Mentha longifolia*	Saudi Arabia	Showed significant antileishmanial activity (161)	[88]
**162**	C_32_H_51_O^5^						
**163**	C_39_H_55_O_7_						
**164**	C_34_H_53_O_6_	colorless powder	*Fusarium* sp.	roots of *Mentha longifolia*	Saudi Arabia	Displayed potent cytotoxic activity towards BT-549 and SKOV-3; Showed potent antileishmanial activities against *L. donovani* promastigotes	[89]
**165**	C_42_H_68_O_7_	white amorphous powder					
Terpenoids Meroterpenoids							
**166**	C_18_H_26_O_5_	colorless crystal	*Guignardia mangiferae* A348	Medicinal Plant *Smilax glabra*	Luofu Mountain Natural Reservation, Guangdong Province, China	Showed weak cytotoxic activities against MCF-7 cell lines(167,169)	[90]
**167**	C_17_H_22_O_4_						
**168**	C_18_H_28_O_5_	white powder					
**169**	C_17_H_24_O_4_						
**170**	C_25_H_30_O_5_	colorless amorphous powder	*Emericella* sp. TJ29	root of the plant *Hypericum perforatum*	the Shennongjia areas of Hubei Province, China	Showed moderate cytotoxic activities against five human tumor cell lines (HL-60, SMMC7721, A549, MCF-7, and SW-480) (172, 173, 175)	[91]
**171**	C_27_H_34_O_6_	white powder					
**172**	C_26_H_32_O_6_	colorless crystals					
**173**	C_25_H_30_O_6_	colorless crystals					
**174**	C_25_H_32_O_6_	white powder					
**175**	C_25_H_28_O_6_	colorless crystals					
**176**	C_31_H_35_O_9_	yellowish oil	*Phyllosticta* sp. J13-2-12Y	leaf of *Acorus tatarinowii*	Guangxi Province, China	Exhibited moderate antimicrobial activities against *Staphylococcus aureus* 209P, *Candida aureus* 209P, and *Candida albicans* FIM709	[92]
**177**	C_31_H_37_O_9_						
**178**	C_31_H_34_O_9_						
**179**	C_27_H_32_O_9_	white powder	Co-culture *Talaromyces purpurogenus* H4 and *Phanerochaete* sp. H2	*Handroanthus impetiginosus*	Alfenas, Minas Gerais, Brazil.	Showed moderate trypanocidal activity against *T. cruzi*	[93]
Lactones							
**180**	C_13_H_22_O_3_	colorless gum	*Talaromyces assiutensis* JTY2	leaf of *Ceriops tagal*	South China Sea, China	Showed moderate cytotoxic activities against three human cancer cell lines (HeLa, MCF-7, and A549)	[94]
**181**	C_21_H_34_O_5_	yellow oil	*Penicillum polonicum*	fruits of *Camptotheca acuminata* Decne	Wuhan, China	Showed effective glucose uptake activity on rat skeletal muscle myoblast L6 (181); Significantly promoted GLUT4 translocation in L6 cells	[95]
**182**	C_16_H_28_O_5_	light red oil					
**183**	C_24_H_33_NO_8_	colorless crystal	*Chaetomium cupreum*	*Anemopsis californica*	New Mexico, U.S.A.	Showed weak antimicrobial activity against *S. aureus*	[96]
**184**			*Xylaria curta* 92092022		Taiwan, China	Showed moderate antimicrobial activities against *Pseudomonas aeruginosa* and *Staphylococcus aureus*; Displayed strongly inhibited lettuce seed germination	[97]
**185**	C_24_H_34_O_5_	white powder	*Lasiodiplodia theobromae* ZJ-HQ1	healthy leaves of the marine mangrove *Acanthus ilicifolius*	South China Sea, China	Exhibited inhibitory effects on lipopolysaccharide-induced nitric oxide production in RAW 264.7 macrophage cells; Showed moderate inhibitory activity against α-glucosidase	[98]
**186**	C_8_H_10_O_3_		*Aspergillus pseudonomiae* J1	*Euphorbia umbellata* (Pax) *Bruyns* (Euphorbiaceae)	Bahia, Brazil	Showed moderate to weak anti-trypanosomal activity	[99]
**187**	C_10_H_12_O_4_						
**188**	C_10_H_12_O_3_						
Anthraquinones, quinones, and related glycosides							
**189**	C_20_H_19_O_7_	Brilliant yellowish oil	*Acremonium vitellinum*	*Acanthus ilicifolius Linn*	Qingdao Province, China	Showed moderate insecticidal activities against the third-instar larvae of Helicoverpa ar-migera	[100]
**190**	C_20_H_16_O_7_	yellow solid					
**191**	C_22_H_21_O_7_						
**192**	C_18_H_18_O_8_Na	yellow amorphous powder	*Phomopsis* sp.	*Paris polyphyllavar*	Yunnan Province, China	Showed significant cytotoxic activities against A549 and PC3 cell lines	[101]
**193**	C_21_H_20_O_10_	red amorphous powder	*Eurotium cristatum* EN-220	*Sargassum thunbergii*	Qingdao Province, China	Showed weak antimicrobial activity against *E. coli* only; Showed moderate fatal activity against brine shrimp larvae	[102]
**194**	C_18_H_15_O_6_	orange yellow powder	*Aspergillus versicolor*	*Halimeda opuntia*	South Sinai, Egypt	Weak inhibitory activity against hepatitis C virus NS3/4A protease	[103]
**195**	C_12_H_11_O_4_	red powder					
**196**	C_16_H_11_O_5_	orange powder					
**197**	C_16_H_21_O_7_	red powder	*Stemphylium globuliferum*	healthy stems of *Mentha pulegium*	Beni Mellal, Morocco	Showed significant inhibition of proliferation of K562 and A549 cells	[104]
Steroids							
**198**	C_27_H_40_O_3_	white crystals	*Phoma* sp. SYSU-SK-7		Guangdong Province, China	Exhibited inhibitory effects on lipopolysaccharide-induced nitric oxide production in RAW 264.7 macrophage cells; Showed moderate inhibitory activity against α-glucosidase	[105]
**199**	C_28_H_41_O_3_	white solid					
**200**	C_38_H_64_O_4_	white amorphous powder	*Fusarium* sp.	*Mentha longifolia*	Egypt	Showed moderate cytotoxic activity against human colorectal cancer cell line HCT 116	[106]
**201**	C_28_H_40_O_4_		*Phomopsis* sp.	*Aconitum carmichaeli*	Huize County, Yunnan Province, China	Showed weak antifungal activities against *C. albicans* and *F. avenaceum*	[107]
**202**	C_30_H_48_O_4_						
Other types of compounds							
**203**	C_20_H_22_O_3_	yellow oil	*Aspergillus* sp.	root of *Tripterygium* *wilfordii*	Wuhan, China	Showed significant inhibition of LPS-induced IL-1β, TNF-α, and NO production in RAW264.7 cells	[108]
**204**	C_24_H_26_O_6_						
**205**	C_24_H_26_O_6_	colorless oil					
**206**	C_23_H_24_O_6_						
**207**	C_23_H_24_O_5_	brown powder	*Aspergillus flavipes* Y-62	stems of plant *Suaeda glauca* Bunge	Zhoushan coast, Zhejiang province, China	Exhibited antimicrobial activities against the Gram-negative pathogens *Pseudomonas aeruginosa* and *Klebsiella pneumoniae*	[109]
**208**	C_23_H_21_O_5_	white powder	*Microsphaeropsis* sp.			Showed effective antimicrobial activities against *B. megaterium* and *E. coli*	[110]
**209**	C_15_H_20_O_2_	brown amorphous powder	*Emericella* sp. XL029	leaf of *Panax* *notoginseng*	Shijiazhuang, Hebei province, China	Showed potent antifungal activities against six tested plant pathogenic fungi (*Rhi-zoctorzia solani*, *Verticillium dahliae Kleb*, *Helminthosporium maydis*, *Fusarium oxysporum*, *Fusarium tricinctum*, and *Botryosphaeria dothidea*)	[111]
**210**	C_16_H_20_O_4_						
**211**	C_18_H_18_O_6_Cl	yellow powder	*Diaporthe* perseae sp.	stem of Chinese mangrove *Pongamia pinnata*	Hainan city, China	Showed significant DPPH and ABTS radical scavenging activities	[112]
**212**	C_7_H_7_O_4_	colorless flake crystal	*Aspergillus tubingensis* DS37	*Decaisnea insignis* (Griff.) Hook. f. and Thomson		Showed significant inhibition activities against *Fusarium graminearum* and *Streptococcus lactis*	[113]
**213**	C_13_H_15_NO_4_Na	amorphous powder	*Nigrospora sphaerica* ZMT05	*Oxya chinensis* *Thunber*	Guangdong Province, China.	Showed significant α-glucosidase inhibitory activity	[114]
**214**	C_9_H_14_O_2_	colorless oil	Co-culture *Aspergillus terreus* EN-539 & *Paecilomyces lilacinus* EN-531	*Laurencia okamurai*	Qingdao, China	Showed weak antimicrobial activities against *Physalospora piricola* and *Staphylococcus aureus*	[115]
**215**	C_11_H_11_BrO_4_	white amorphous powder	*Lachnum palmae*	*Przewalskia tangutica*		Exhibited potent to weak antimicrobial activities against *Cryptococcus neoformans*, *Penicillium sp.*, *Candida albicans*, *Bacillus subtilis*, *and Staphy-lococcus aureus* (215–220); Showed moderate inhibitory effects on NO production in LPS-induced RAW 264.7 cells (215,219)	[116]
**216**							
**217**	C_12_H_13_BrO_4_						
**218**	C_10_H_9_BrO_4_						
**219**	C_11_H_11_BrO_5_						
**220**	C_11_H_11_ClO_4_						

**Table 3:** Culture conditions and yields of bioactive secondary metabolites produced by endophytic fungi.

No.	Endophytic Fungus	Host Plant	Culture Conditions	Secondary Metabolites	Yield	Ref.
1	*Hansfordia biophila*	*Hedychium acuminatum* Roscoe	Inoculated in potato glucose broth (PDB) medium and shaken at 120 rpm at 25 °C for 7 days.	Tannin	41.6 μm·mL^−1^	[121]
2	*Aspergillus terreus*	*Ficus elastica*	Inoculated into PDB medium and incubated at 30 °C for 20 days on a rotatory shaker incubator at 140 rpm.	Camptothecin	320 μg/L	[122]
3	*Guignardia mangiferae* HAA11	*Taxus x media*	Inoculated into (PDB) medium and incubated at 200 rpm at 28 °C for 5 days.	Paclitaxel	720 ng/L	[123]
4	*Papulasora* sp.S6	*Phellodendron amurense* Rupr	Mutagenesis by UV, X-ray rays, and NaNO_2_, inoculated in PDB medium, and shaken at 100 rpm at 28 °C for 7 days.	Berberine	12.28 mg/L	[124]
5	*Actinoplanes teichomyceticus*		Improvement of the output of teicoplanin by genome shuffling; Inoculated teicoplanin medium and cultured at 28 °C for 15–20 days.	Teicoplanin	3016 μm·mL^−1^	[125]
6	*Phialocephala fortinii*	*Podophyllum peltatum*	Inoculated in Sabouraud’s dextrose agar (SDA) and cultured at 23 °C for 4–6 weeks.	Podophyllotoxin	189 µg/L	[126]
7	*Entrophospora infrequens* *RJMEF001*	*Nothapodytes foetida*	Inoculated into wheat bran containing Sabouraud’s broth, and incubation was carried out at 28 ± 2 °C for 28 days.	Camptothecin	503 ± 25 μg/100 g dry cell mass (in Sabouraud broth)	[127]
8	*Epicoccum nigrum* SZMC 23769	*Hypericum perforatum*	Fungal isolates were grown in potato dextrose broth (PDB) for 7 days at 25 °C.	Hypericin, Emodin	117.1 μg/mL, 87.7 μg/mL	[128]

## Data Availability

Not applicable.
